# Separase Is Required for Homolog and Sister Disjunction during *Drosophila melanogaster* Male Meiosis, but Not for Biorientation of Sister Centromeres

**DOI:** 10.1371/journal.pgen.1005996

**Published:** 2016-04-27

**Authors:** Ariane C. Blattner, Soumya Chaurasia, Bruce D. McKee, Christian F. Lehner

**Affiliations:** 1 Institute of Molecular Life Sciences (IMLS), University of Zurich, Zurich, Switzerland; 2 Department of Biochemistry, Cellular and Molecular Biology (BCMB), University of Tennessee, Knoxville, Tennessee, United States of America; Massachusetts Institute of Technology, UNITED STATES

## Abstract

Spatially controlled release of sister chromatid cohesion during progression through the meiotic divisions is of paramount importance for error-free chromosome segregation during meiosis. Cohesion is mediated by the cohesin protein complex and cleavage of one of its subunits by the endoprotease separase removes cohesin first from chromosome arms during exit from meiosis I and later from the pericentromeric region during exit from meiosis II. At the onset of the meiotic divisions, cohesin has also been proposed to be present within the centromeric region for the unification of sister centromeres into a single functional entity, allowing bipolar orientation of paired homologs within the meiosis I spindle. Separase-mediated removal of centromeric cohesin during exit from meiosis I might explain sister centromere individualization which is essential for subsequent biorientation of sister centromeres during meiosis II. To characterize a potential involvement of separase in sister centromere individualization before meiosis II, we have studied meiosis in *Drosophila melanogaster* males where homologs are not paired in the canonical manner. Meiosis does not include meiotic recombination and synaptonemal complex formation in these males. Instead, an alternative homolog conjunction system keeps homologous chromosomes in pairs. Using independent strategies for spermatocyte-specific depletion of separase complex subunits in combination with time-lapse imaging, we demonstrate that separase is required for the inactivation of this alternative conjunction at anaphase I onset. Mutations that abolish alternative homolog conjunction therefore result in random segregation of univalents during meiosis I also after separase depletion. Interestingly, these univalents become bioriented during meiosis II, suggesting that sister centromere individualization before meiosis II does not require separase.

## Author Summary

Sexual reproduction depends on meiosis, a special cell division that occurs in two steps, meiosis I and II. Meiosis is distinct in males and females that produce two very different forms of compatible gametes, the sperm and egg, respectively. In the fly *Drosophila melanogaster*, sex-specific differences are pronounced. While pairing of homologous chromosomes into bivalents before the first meiotic division proceeds in a canonical manner in females, males use an alternative system. After production of bivalents and their biorientation in the meiosis I spindle, they need to be split up again in both sexes so that chromosomes can be segregated to opposite spindle poles during anaphase I. Here we demonstrate that separase, a protease that separates canonical bivalents by cleavage of a cohesin subunit, is also required during male meiosis I, even though alternative homolog conjunction does not depend on cohesin. Moreover, our results suggest that the separation of sister centromeres into functionally distinct units that has to occur between the first and second meiotic division does not depend on separase. Centromeric cohesin might therefore either not enforce the crucial sister centromere coorientation during meiosis I, or be removed in a separase-independent manner in preparation for meiosis II.

## Introduction

After their production during S phase, sister chromatids remain paired. This sister chromatid cohesion is crucial for proper bipolar chromosome orientation within mitotic spindles during early M phase. Sister chromatid cohesion is maintained primarily by cohesin, a protein complex composed of an SMC1/3 heterodimer and accessory subunits including an α-kleisin protein [1, 2]. However, during late metaphase after biorientation of all chromosomes within the spindle, cohesion between sister chromatids needs to be released for chromosome segregation during anaphase. This cohesion release depends on separase, an endoprotease which specifically cleaves α-kleisin just before the metaphase to anaphase transition [1–5]. Loss of cohesin or separase function results in chromosome segregation errors due to premature separation of sister chromatids or failure of their separation, respectively.

In comparison to mitosis, chromosome segregation during meiosis is more elaborate [6]. After pre-meiotic S phase, homologous chromosomes pair up and form bivalents. Maintenance of homologous chromosome pairs usually depends on chiasmata generated by meiotic recombination. Importantly, the two sister kinetochores in each chromosome are also united into a functional unit and co-oriented during the first meiotic division [7–12]. This allows bipolar orientation of bivalents in the meiosis I spindle. Separase-mediated release of cohesin from chromosome arms during late metaphase I permits terminalization of chiasmata and chromosome segregation during anaphase I [13–17]. Importantly, however, release of pericentromeric cohesin is prevented during meiosis I [18–24]. This keeps sister kinetochores paired. Moreover, since sister kinetochores regain functional individuality, they become bioriented within meiosis II spindles and segregate to opposite spindle poles after separase-mediated destruction of pericentromeric cohesin during late metaphase II [25–28]. Faithful chromosome segregation during meiosis therefore relies on the unique functional unification of sister kinetochores during meiosis I, in combination with temporally controlled release of arm and pericentromeric cohesion during meiosis I and II, respectively.

Interestingly, the success of meiosis depends apparently not only on the two chromosomal cohesin populations in arm and pericentromeric regions, but also on yet another, functionally distinct cohesin pool acting within the centromere. Centromeric cohesin was proposed to be present and required exclusively before meiosis I in fission yeast for the functional unification and co-orientation of the two sister kinetochores [9, 26, 29, 30]. Analyses in mouse oocytes [31, 32] and plants [33] have provided further support for a functional unification of sister kinetochores by meiosis I-specific centromeric cohesin. The molecular mechanisms that establish and inactivate centromeric cohesin before meiosis I and II, respectively, are poorly understood. Spo13, Moa1 and Meikin, which appear to provide a similar function required for sister kinetochore co-orientation during meiosis I in budding yeast, fission yeast and vertebrates, respectively, might be involved in the generation of centromeric cohesion [11]. Moreover, destruction of centromeric cohesin during exit from meiosis I by separase seems to be an obvious, highly probable mechanism for sister kinetochore individualization before meiosis II.

Beyond unknown aspects of canonical meiosis, additional issues remain to be clarified in the context of derived meiotic variants. In Drosophila males, pairing and physical linkage of homologous chromosomes involves neither meiotic recombination nor synaptonemal complex formation [34]. Several components of an alternative homolog conjunction system have been identified genetically. Mutations in *modifier of mdg4 in meiosis (mnm*) and *stromalin in meiosis*/*SA-2 (snm)* result in random segregation of homologs in meiosis I [35]. Cytological analyses have revealed homolog conjunction defects in the mutants [35]. The proteins MNM and SNM accumulate exclusively in spermatocytes. At the start of meiosis, they are recruited prominently to a spot on the X-Y chromosome bivalent. Although the two sex chromosomes are strongly heteromorphic in Drosophila, they both contain a locus with an rDNA gene cluster and 240 bp repeats within the intergenic sequences. These repeats were shown to be required and sufficient for X-Y pairing [36]. Cytology has revealed co-localization of these repeats with MNM and SNM [35, 37]. In addition, weak MNM/SNM spots were also observed on autosomes. The molecular details of MNM and SNM recruitment to meiotic chromosomes remain to be analyzed. MNM contains a unique C-terminal FLYWCH Zn-finger domain and an N-terminal BTB/POZ domain that is also present on many other *mod(mdg4)* isoforms [35]. SNM is a distant relative of the stromalins which are known to be cohesin subunits [35]. Beyond *mnm* and *snm*, homolog conjunction in Drosophila males also requires *teflon (tef)* which codes for a protein with three C2H2-type zinc fingers and unknown localization during meiosis [38]. *tef* expression does not appear to be meiosis-specific and it is only required for autosome but not X-Y conjunction [38].

The fact that homologous chromosomes in Drosophila males are not linked by chiasmata and arm cohesion raises the question of how homologs are separated during meiosis I. In principle, separase activity might inactivate the alternative homolog conjunction system before anaphase I and thus cause homolog separation as in canonical meiosis. However, separase appears to target primarily cohesin which does not appear to be involved in alternative homolog conjunction. Hence, a separase-independent mechanism remains a possibility as well. Therefore, we evaluated the role of separase during Drosophila male meiosis. Moreover, the possibility of separase-independent chromosome segregation during Drosophila male meiosis I appeared to offer opportunities for confirmation that separase is actually required for sister kinetochore individualization during exit from meiosis I, since in this case normal chromosome segregation during meiosis I would be predicted to be followed by a failure of sister kinetochore biorientation during meiosis II in the absence of separase function. The apparent absence of a Drosophila *rec8* homolog [39, 40] provided yet another reason for our interest in meiotic separase functions. A meiosis-specific Rec8 alpha-kleisin has been shown to be absolutely required for protection of pericentromeric cohesion from separase cleavage during meiosis I in a wide range of species [15, 21, 23, 24, 26, 33, 41]. Its putative absence in Drosophila further emphasizes the non-canonical nature of its meiosis, making meiotic separase functions highly unpredictable in this species where potentially not just chromosome separation during meiosis I but also during meiosis II might be achieved in a separase-independent fashion.

To evaluate meiotic functions of separase, we developed approaches for separase inhibition specifically during male meiosis. Thereby we were able to demonstrate that the inactivation of the alternative homolog conjunction system before anaphase I onset is entirely dependent on separase. Moreover, the fact that alternative conjunction in Drosophila males can be eliminated by specific mutations that do not affect sister chromatid cohesion also allowed us to address whether sister kinetochore individualization after metaphase I depends on separase. Interestingly, we find that sister kinetochore biorientation during meiosis II appears to be independent of separase.

## Results

### Separase is required for normal chromosome segregation during Drosophila male meiosis

Drosophila Separase (SSE) does not have a large N-terminal regulatory region as typically observed in other species [42]. However, it forms a complex with the product of the *three rows (thr)* gene which appears to have resulted from a separase gene split during Drosophila evolution [43, 44]. Moreover, the product of the *pimples (pim)* gene also needs to accumulate during interphase and join the SSE-THR complex for eventual separase function during mitosis [42, 45]. Therefore, embryos lacking *pim* function zygotically also display a separase loss-of-function phenotype. This mutant phenotype does not reveal that PIM actually also has an additional, separase-inhibitory role. However, PIM is known to be the Drosophila securin homolog that prevents premature separase activity until late metaphase [46, 47]. While PIM is required initially for separase complex formation, it has to be degraded again eventually during late metaphase via activation of the ubiquitin ligase APC/C. The meiotic function of *Sse*, *thr* and *pim* cannot be studied in zygotic null mutants. They do not develop to the developmental stages where progression through meiosis starts because of sister chromatid separation failure during the earlier mitotic divisions [42, 45, 48]. To investigate meiotic functions, we applied transgenic RNAi expressed specifically in spermatocytes using the *bam-GAL4-VP16* (*bG*) driver. Knock down of *thr* was found to be most effective, causing male sterility (S1 Table).

**Fig 1:**
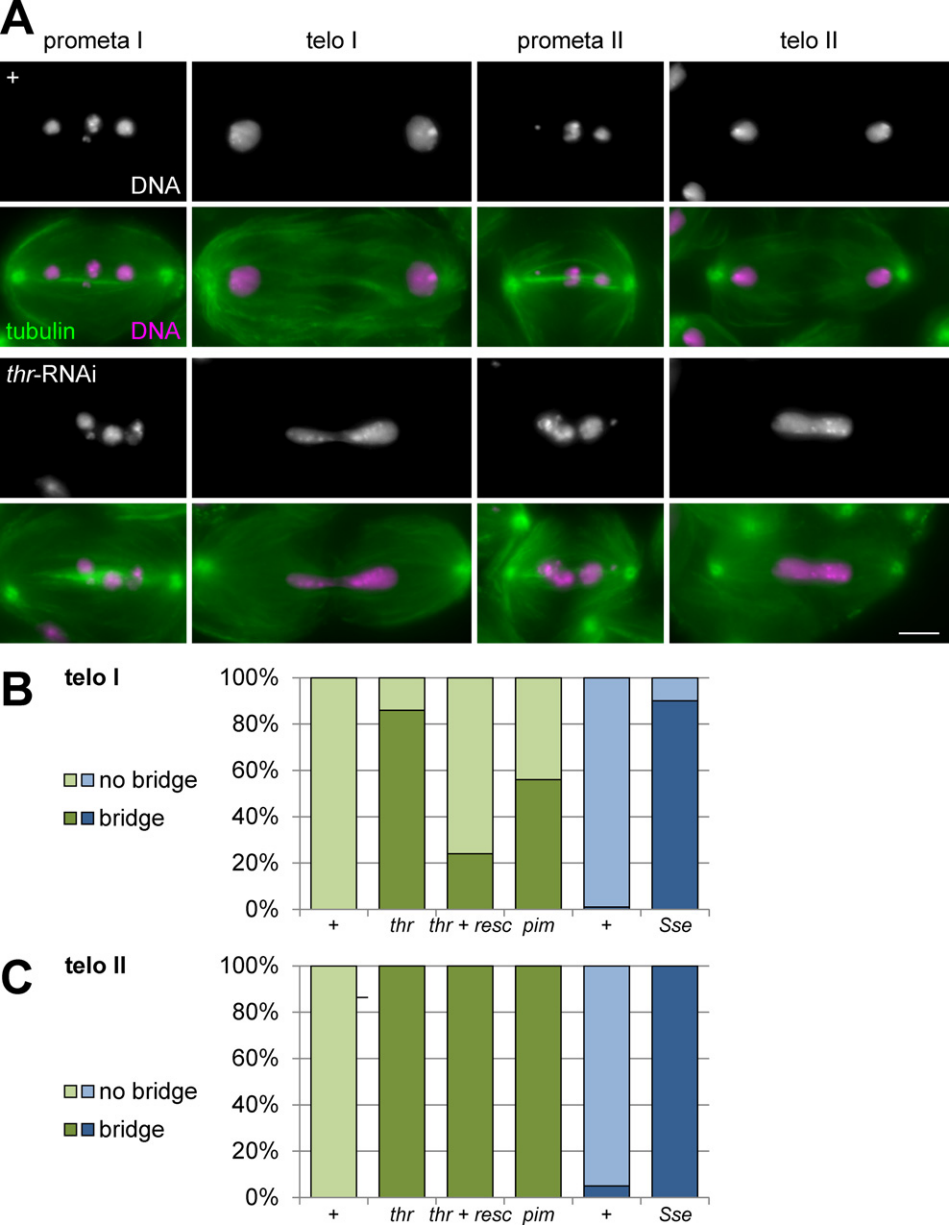
Depletion of separase complex subunits results in chromosome bridges during telophase I and II. (A) Testes were isolated from males without (+) or with spermatocyte-specific THR depletion by transgenic RNAi (*thr*-RNAi). Squash preparations were labeled with anti-tubulin (tubulin) and a DNA stain (DNA). Single spermatocytes at the indicated meiotic stages are displayed. Scale bar = 5 μm. (B) Bars represent the percentage of spermatocytes in telophase I that displayed a chromosome bridge. Testes analyzed were from males without (+) or with spermatocyte-specific THR (*thr*) or PIM (*pim*) depletion by RNAi, as well from males without (+) or with spermatocyte-specific SSE depletion by deGradFP (*Sse*). In case of THR depletion, we also analyzed testes, in which transgenic RNAi was accompanied by expression of an RNAi-resistant *thr* transgene (*thr* + resc). (C) Bars represent the percentage of cysts with spermatocytes that displayed chromosome bridges during telophase II. The same genotypes as in (B) were analyzed. Precise genotype descriptions and the number of samples analyzed for the bar diagrams (B, at least 145 telophase I figures per genotype; C, at least 10 telophase II cysts) are given in S1 Table.

To characterize the effects of THR depletion at a cellular level, we analyzed testis squash preparations after staining with a DNA stain and anti-tubulin. Anti-tubulin labeling facilitates identification of meiotic cells and discrimination of meiosis I and II also when chromosome segregation is abnormal. Cells during the meiotic divisions have spindles that change in a characteristic manner during progression through these divisions. During late anaphase and telophase for example, formation of a prominent central spindle occurs. Anti-tubulin labeling also revealed the overall cell size which is halved by cytokinesis first during meiosis I and once again during meiosis II. Conversely, cell number per cyst increases and also provided information whether cells are in meiosis I or II. Preparations with testes from *bG* males with and without *UASt-thr*^*RNAi*^ transgene displayed normal prometaphase I figures (Fig 1A).

Up to four DNA masses could be resolved corresponding to the bivalents with the sex chromosomes (XY) and the three autosomes (2^nd^, 3^rd^, and small 4^th^ chromosome). In the control preparations, telophase I figures and the figures from prometaphase II and telophase II were normal as expected (Fig 1A). Telophase cells contained two round daughter nuclei of comparable size close to the two spindle poles. In contrast, after THR depletion 86% of the telophase I figures were clearly abnormal (Fig 1A and 1B). Bi-lobed DNA staining with a connecting chromosome bridge was observed (67%), as well as cases with a single DNA mass that was no longer bi-lobed (19%). Subsequent meiotic stages were also abnormal after THR depletion (Fig 1A). Prometaphase II cells contained highly variable amounts of chromatin. Moreover, chromosome separation failure was again apparent during telophase II in 100% of the cysts (Fig 1C).

Time-lapse analysis of progression through the meiotic divisions without and with THR depletion (S1 Movie and S2 Movie) fully confirmed the findings from squash preparations. THR depletion did not affect meiosis I up to anaphase onset. However, chromosome separation during anaphase I did not succeed. Subsequent cytokinesis was irregular as well, producing a pair with a nucleate and an anucleate cell in some cases, or cutting through the undivided mass of chromatin in other cases. As a result, meiosis II spindles were often abnormal. But even in cases with normal meiosis II spindles, chromosome separation during anaphase II never occurred normally. Time-lapse imaging also revealed that THR depletion did not severely affect the dynamics of progression through meiosis I. However, the number of our movies that start before nuclear envelope breakdown in meiosis I is low (only two cysts from independent preparations) because time-lapse imaging was usually started only after finding a cyst within the testis preparation which already had initiated meiosis I. Starting time-lapse imaging earlier at a stage where cysts are still in the long premeiotic G2 phase allows analysis of progression through meiosis only in very rare fortuitous cases because cyst viability deteriorates in most of the cases before entry into meiosis. In the two completely tracked THR depleted meiotic cysts, the duration of prometaphase I, metaphase I, and anaphase I was 12/18, 12/16, and 11/12 minutes, respectively. The average duration of these meiosis I phases in controls was 12.3 ± 1.8, 13.3 ± 3.7, and 10.7 ± 1.2 minutes respectively (± s.d.; n = 6 cysts from independent preparations). Additional movies allowing determination of the duration of metaphase I (n = 2) and anaphase I (n = 4) after THR depletion provided further support for our conclusion that the temporal dynamics of congression of bivalents and onset of anaphase during meiosis I were not severely altered by THR depletion, although subtle effects cannot be excluded. As a result of the chromosome separation defects caused by THR depletion during meiosis I, meiosis II was severely affected in various variable ways and therefore, analysis of temporal dynamics during interkinesis and meiosis II after THR depletion was not attempted.

As *bG*-directed expression starts already during the final mitotic division cycles that generate the cysts of 16 interconnected spermatocytes, the meiotic defects described above might arise in principle as secondary consequences from earlier division abnormalities. However, several observations indicated that the cysts entering into meiosis after *bG*-directed THR depletion were normal. Cysts still comprised 16 cells as expected. Moreover, the number of centromeres detected during meiosis indicated that all spermatocytes were euploid. FISH with X and Y probes, as well as MNM/SNM immunolocalization also confirmed euploidy (see below). However, testes of *bG* males with *UASt-thr*^*RNAi*^ were smaller than those of control males. Therefore, some germline stem cell or gonial cyst depletion might occur as a result of leaky expression during earlier stages.

To rule out off-target effects, we introduced into the males with *bG* and *UASt-thr*^*RNAi*^ also an *UASt-thr*^*Rr*^ transgene predicted to be RNAi-resistant as a result of silent mutations. While the RNAi-resistant transgene was unable to restore a completely normal meiosis, it reduced the frequency of telophase I abnormalities fourfold (Fig 1B). Moreover, the residual chromosome bridges during telophase I were less massive and less stable when the RNAi-resistant transgene was present (S1 Fig).

For further confirmation that the abnormalities resulting after THR depletion reflect a loss of separase function, we depleted other separase complex subunits. Analogous *bG* directed expression of *UASt-Sse*^*RNAi*^ and *UASt-pim*^*RNAi*^ resulted in no or milder abnormalities, respectively. Abnormalities after PIM depletion were also first observed during telophase I, as in case of THR depletion. Chromosome bridges were apparent in 56% of the telophase I figures (Fig 1B) and all cysts during telophase II also displayed chromosome bridges. To achieve SSE depletion, we applied an alternative method, deGradFP which allows regulated proteolytic degradation of GFP fusion proteins [49]. Therefore, we first generated a line in which the lethality associated with hemizygosity for an *Sse* null mutation (*Sse*^*13m*^/*Df(3L)SseA*) was prevented by a transgene driving expression of EGFP-SSE under control of the *Sse* cis-regulatory region (*gEGFP-Sse*). The resulting flies were also fertile (S1 Table). However, introduction of an additional transgene directing *NSlmb-vhh-GFP4* expression by the *bam* regulatory region led to complete sterility (S1 Table). NSlmb-vhh-GFP4 has been shown to result in polyubiquitination and proteasomal degradation of GFP fusion proteins [49]. Analyses of testis squash preparations revealed that SSE depletion by deGradFP resulted in abnormalities that were indistinguishable from those caused by *thr*-RNAi (Figs 1B, 1C and S2).

In summary, spermatocyte-specific depletion of the three separase complex subunits, THR, PIM or SSE, with different strategies (transgenic RNAi and deGradFP) was found to cause the same specific defect during meiosis. Importantly, defects start already during anaphase I. Although the degree of interference was lower in case of PIM, presumably as a result of incomplete depletion, chromosome segregation during anaphase I was affected in each case. We conclude that separase function is required for chromosome segregation during both meiotic divisions in Drosophila males.

### Separase is required for the release of alternative homolog conjunction proteins during meiosis I

**Fig 2:**
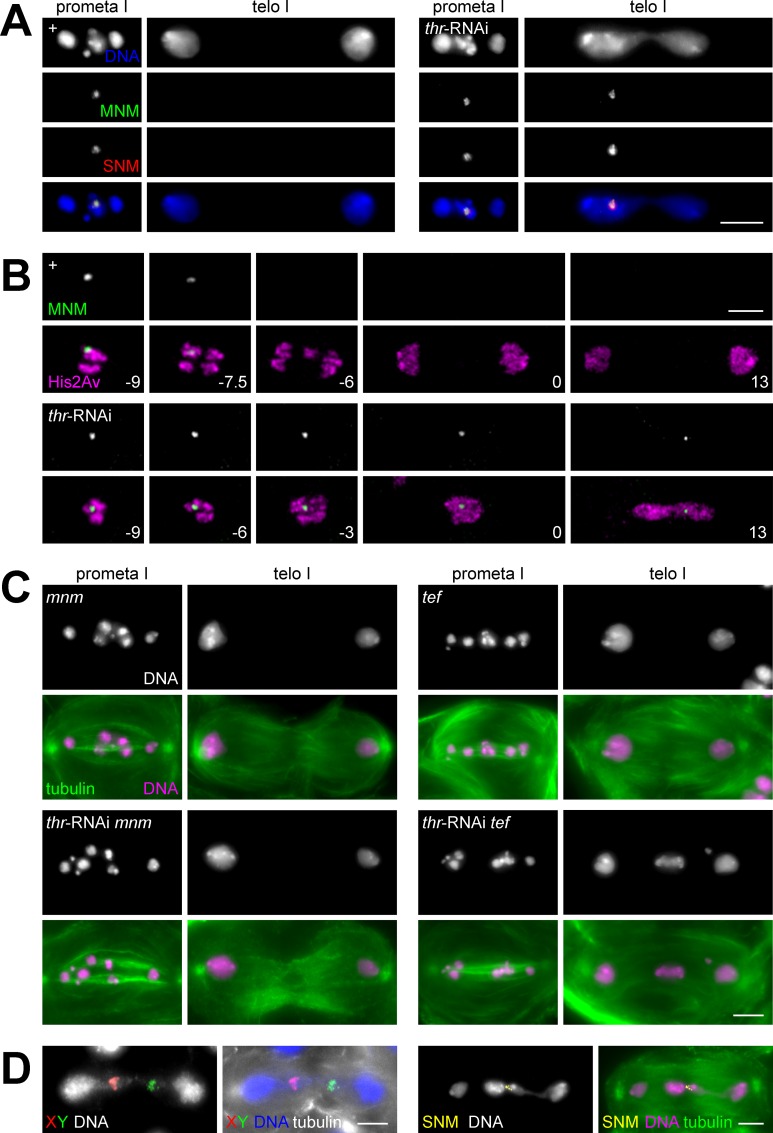
THR depletion causes failure of chromosomal MNM/SNM release and homolog separation during meiosis I. (A) Testes were isolated from *mnm-EGFP* males without (+) or with spermatocyte-specific THR depletion by transgenic RNAi (*thr*-RNAi). Squash preparations were labeled with anti-SNM (SNM) and a DNA stain (DNA). Single spermatocytes at the indicated meiotic stages are displayed. Scale bar = 5 μm. (B) Still frames after time-lapse imaging of progression through meiosis I with testes expressing *mnm-EGFP* (MNM) and *His2Av-mRFP* (His2Av). Moreover, testes were from males without (+) or with spermatocyte-specific THR depletion by transgenic RNAi (*thr*-RNAi). Time in minutes is indicated in the lower right corner of the merged images with t = 0 representing the time when chromatin decondensation had progressed to the point where chromosome arms could no longer be resolved individually. In the control (+), the last metaphase I frame was at -9 minutes and the MNM-EGFP dot disappeared within the first 3 minutes of anaphase I. After *thr*-RNAi, the MNM-EGFP dot persisted throughout exit from meiosis I. Scale bar = 5 μm. (C) Squash preparations were labeled with anti-tubulin (tubulin) and a DNA stain (DNA). The images on the left side are from *mnm* mutant males without (*mnm*) or with spermatocyte-specific THR depletion by transgenic RNAi (*thr*-RNAi *mnm*). The images on the right side are from *tef* mutant males without (*tef*) or with spermatocyte-specific THR depletion by transgenic RNAi (*thr*-RNAi *tef*). Single spermatocytes at the indicated meiotic stages are displayed. Scale bar = 5 μm. (D) Squash preparations of testis from *tef* mutant males with *thr*-RNAi were labeled with anti-tubulin (tubulin) and a DNA stain (DNA). In addition, FISH with a red fluorescent X chromosome probe (X) and a green fluorescent Y chromosome probe (Y) was performed in case of the images on the left side, and anti-SNM (SNM) labeling in case of the images on the right side. Scale bar = 5 μm.

To determine whether separase is required for the removal of alternative homolog conjunction proteins before chromosome segregation during meiosis I in Drosophila males, we analyzed the effects of THR depletion on the subcellular localization of MNM and SNM with the help of a fully functional *mnm-EGFP* transgene [35] and anti-SNM antibodies [35]. As previously described [35], co-localized MNM-EGFP and anti-SNM signals were detected primarily in a highly prominent spot on the XY bivalent during prometaphase and metaphase of meiosis I (Fig 2A).

In contrast, no MNM-EGFP/anti-SNM spot was detected during telophase I (n = 47) (Fig 2A) and later meiotic stages in control testis where meiosis I is normal [35]. However, after THR depletion, MNM-EGFP/anti-SNM spots were not only present early during meiosis I, but also during telophase I (n = 209 spermatocytes) (Fig 2A) and all subsequent stages up to the postmeiotic stages of sperm tail elongation (S3 Fig). In telophase I cells with chromosome bridges, the MNM-EGFP/anti-SNM spots were usually associated with the bridge (94%). Telophase I cells without obvious chromosome bridges (6%) also had always an MNM-EGFP/anti-SNM spot in one of the two daughter nuclei. For confirmation, we also performed time-lapse imaging with testis expressing MNM-EGFP and a red fluorescent histone (Fig 2B, S3 Movie and S4 Movie). In control spermatocytes, the MNM-EGFP spot was observed to disappear during early anaphase I within 2–3 minutes (n = 18 spermatocytes). In contrast, after THR depletion chromosome separation failure was accompanied by persistence of the MNM-EGFP spot during exit from meiosis I (n = 76 spermatocytes).

By anti-SNM staining of testis squash preparations, we evaluated whether PIM depletion by RNAi or SSE depletion by deGradFP also resulted in SNM perdurance beyond anaphase I (S4 Fig). These stainings clearly revealed anti-SNM signals during telophase I and meiosis II in spermatocytes depleted for these other separase complex subunits. We conclude that separase function is required for the release of alternative homolog conjunction proteins from chromosomes during progression through meiosis I.

**Fig 3:**
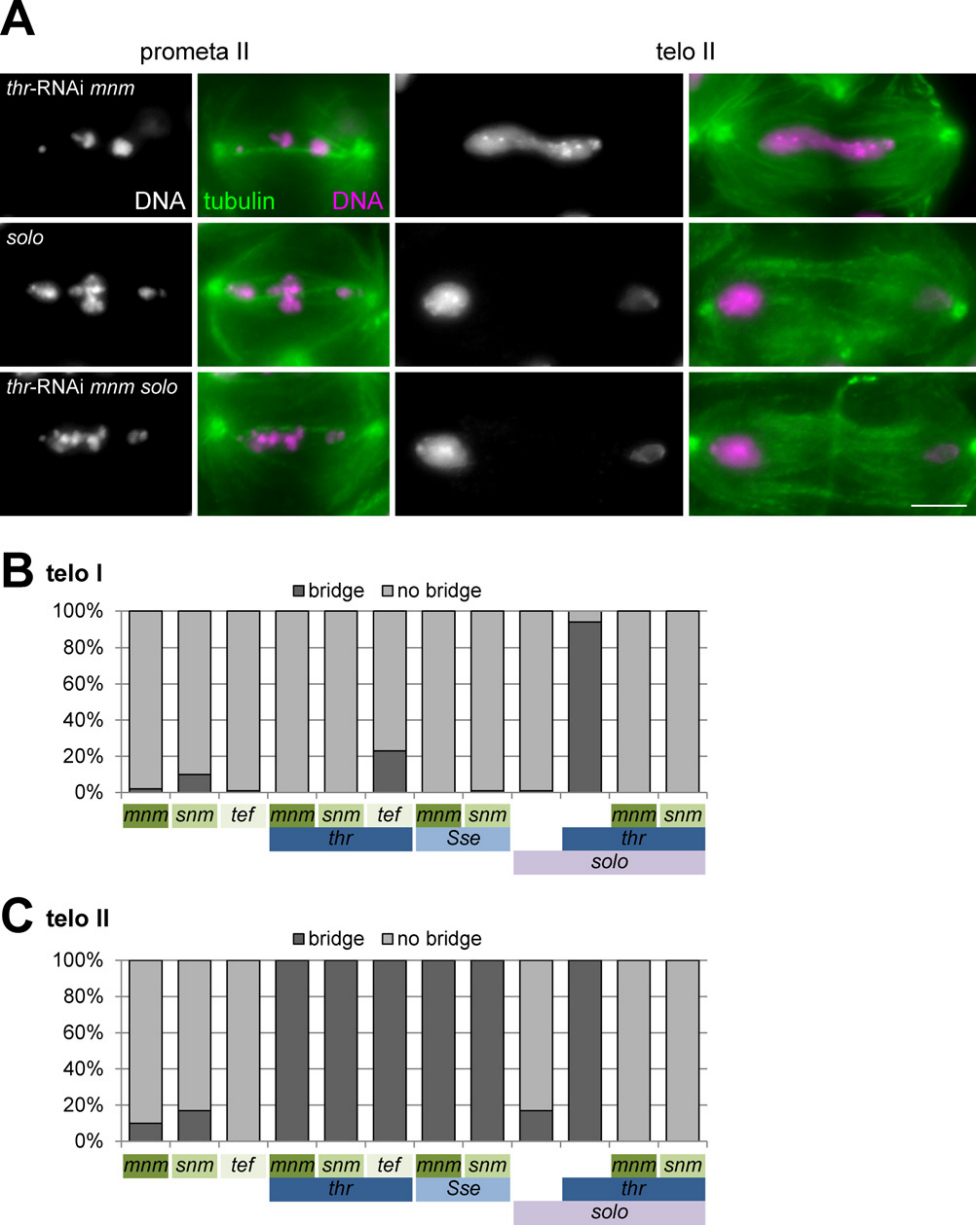
THR and SSE are required for sister chromatid separation during meiosis II. (A) Testis squash preparations were labeled with anti-tubulin (tubulin) and a DNA stain (DNA). Single spermatocytes at the indicated stages during meiosis II are displayed. Testes were isolated from *mnm* mutant males with spermatocyte-specific THR depletion (top row, *thr*-RNAi *mnm*), from *solo* mutant males (middle row, *solo*), and from *mnm solo* double mutant males with spermatocyte-specific THR depletion (bottom row, *thr*-RNAi *mnm solo*). Mutations in *solo* but not *mnm* suppress chromosome bridges induced by THR depletion during meiosis II, indicating that these bridges represent stretched univalents. Scale bar = 5 μm. (B) Bars represent the percentage of spermatocytes in telophase I that displayed a chromosome bridge. Testes analyzed were from males with the genotypes indicated below the bars. *thr* indicates spermatocyte-specific THR depletion by RNAi, and *Sse* spermatocyte-specific depletion of SSE by deGradFP. *mnm*, *snm*, *tef*, and *solo* indicate loss-of-function mutants. (C) Bars represent the percentage of cysts with spermatocytes that displayed chromosome bridges during telophase II. The same genotypes as in (B) were analyzed. Precise genotype descriptions and the number of samples analyzed for the bar diagrams (B, at least 82 telophase I figures per genotype; C, at least 5 telophase II cysts) are given in S1 Table.

The observed failure of MNM and SNM release from chromosomes at anaphase I onset after THR depletion is likely responsible for the associated failure of homolog separation during meiosis I. Accordingly, when alternative homolog conjunction fails to be established, THR depletion is no longer expected to cause chromosome bridging during telophase I. To evaluate this prediction, we depleted THR in *mnm* and *snm* mutants. However, we first confirmed that mutations in *mnm* and *snm* do not cause chromosome bridges during telophase I independent of THR depletion. Consistent with earlier reports [35], instead of four bivalents as in wild type, we observed up to eight chromatin masses during prometaphase I in *mnm* and *snm* mutants (Figs 2C and S5), indicating the known defect in homolog conjunction. In addition, daughter nuclei were often unequal in size and DNA content (Figs 2C and S5), reflecting the known random distribution of chromosomes during meiosis I [35]. Chromosome bridges were very rare in telophase I (Fig 3B).

The few bridge-like structures in telophase I that were observed in *mnm* and *snm* mutants presumably represent univalents lagging in the division plane rather than stretched bivalents [35]. The low frequency of chromosome bridges during meiosis I in *mnm* and *snm* mutants also confirms that co-orientation of sister kinetochores during meiosis I does not depend on homolog conjunction during male meiosis [35]. THR depletion in *mnm* mutants did not induce any phenotypic change during meiosis I (Figs 2C and S5). Importantly, it did not cause chromosome bridges during telophase I (Figs 2C and 3B), in striking contrast to the effect of THR depletion in spermatocytes with functional alternative homolog conjunction (Fig 1). Moreover, THR depletion did also not result in chromosome bridges during telophase I in *snm* mutants (Figs 3B and S5). In addition, SSE depletion by deGradFP did also no longer cause chromosome bridges during telophase I when performed in *mnm* or *snm* mutants (Figs 3B and S5). In summary, the fact that *mnm* and *snm* mutations, which abolish the alternative homolog conjunction in male meiosis, suppress chromosome bridge formation during telophase I in spermatocytes progressing through meiosis in the absence of separase function indicates that separase is required for the resolution of homolog conjunction during meiosis I.

Our conclusion that absence of separase function during Drosophila male meiosis I results in a specific failure of homolog separation is further supported by analyses with an additional mutant. *tef* mutants were chosen because alternative homolog conjunction is also defective in these mutants but not completely. While autosomes fail to pair into bivalents, formation of the X-Y bivalent is not affected [38]. THR depletion in *tef* mutants, therefore, is predicted to result in a chromosome bridge during telophase I that always represents a stretched XY bivalent, if resolution of homolog conjunction during male meiosis I requires separase function. As in case of *nmn* and *snm* mutants, chromosome bridges during telophase I in *tef* mutants with undisturbed separase function (Figs 2C and 3B) were infrequent and presumably represent occasional lagging autosomes. THR depletion in *tef* mutants resulted in an increased frequency of chromosome bridges during telophase I (Figs 2C and 3B). However, these bridges were fewer and less massive compared to those induced by THR depletion in spermatocytes with entirely normal homolog conjunction (Figs 1, 2A and 2B), consistent with the notion that only the XY bivalent forms bridges in *tef* mutants, while all bivalents form bridges in case of normal homolog conjunction. To demonstrate directly that the bridges resulting from THR depletion in *tef* mutants represent stretched XY bivalents, we performed fluorescence in situ hybridization (FISH) with a red fluorescent probe for the X and a green fluorescent probe for the Y chromosome (Fig 2D). After THR depletion in *tef* mutants, the red and green signals were invariably observed on the bridge (Fig 2D). Moreover, anti-SNM staining also resulted in a dot on the non-segregating DNA mass resulting after THR depletion in *tef* mutants (Fig 2D).

FISH was also applied to analyze the segregation of the X and Y chromosome after THR and SSE depletion in spermatocytes with normal homolog conjunction, and also in *mnm* and *snm* mutants after SSE depletion (S6 Fig). These analyses confirmed that disjoining of X and Y was inhibited in the absence of separase function and that the suppression of chromosome bridges resulting from a lack of separase function by inactivation of the alternative homolog conjunction system was paralleled by random segregation of X and Y during meiosis I. Finally, live imaging of progression through meiosis in spermatocytes with green fluorescent centromeres and red fluorescent chromosomes indicated that THR depletion causes chromosome separation failure directly and not indirectly via impairment of spindle or kinetochore function or premature exit from meiosis I (S7 Fig and S5 and S6 Movies).

### Separase is required for sister chromatid separation in meiosis II

While mutations inactivating the alternative homolog conjunction system during Drosophila male meiosis very effectively suppressed chromosome bridges during telophase I after THR depletion (Figs 2C and 3B), they completely failed to do so during telophase II (Fig 3A and 3C). These telophase chromosome bridges presumably reflect a failure to separate sister centromeres during meiosis II in the absence of separase function. To confirm this notion, we performed experiments with mutations in *sisters on the loose (solo)*. SOLO has no sequence homology to known proteins [50, 51]. However, analysis of the *solo* mutant phenotype revealed that it provides a function analogous to that of Rec8 in other eukaryotes. Rec8 is a meiosis-specific α-kleisin. Pericentric cohesin with Rec8 instead of the non-meiosis-specific Rad21 α-kleisin is protected from separase-dependent cleavage during meiosis I but no longer during meiosis II [6]. The Drosophila genome does not contain an obvious Rec8 homolog. But SOLO expression is also meiosis-specific [50, 51]. It interacts physically and functionally with the SMC1 core cohesin subunit and it is present at meiotic centromeres until anaphase II. Mutations in *solo* result in premature separation of sister chromatids. The chromosome bridges observed during telophase II after THR depletion in *mnm* mutants are therefore predicted to be abolished when the spermatocytes also lack *solo* function, if the bridges reflect stretched sister chromatids. Indeed, chromosome bridges during telophase II were not only absent in *solo* single mutants but also after THR depletion in *solo mnm* double mutants (Fig 3A and 3C). Chromosome bridges during telophase II were also missing after THR depletion in *solo snm* double mutants (Figs 3C and S8). These results demonstrate that Separase is required for resolution of sister chromatids in meiosis II.

We point out that mutations in *solo*, in contrast to mutations in *mnm* and *snm*, did not abolish the THR depletion-induced chromosome bridges during meiosis I (Figs 3B and S9), indicating that the alternative homolog conjunction system functions independent of sister chromatid cohesion. Consistent with this conclusion, earlier data reported as unpublished [50] indicated that MNM and SNM are present on bivalents in *solo* mutants where the large majority of bivalents also remain intact through metaphase I. Anti-SNM staining revealed that the prominent SNM dot on the XY bivalent cannot be detected any longer in telophase I and subsequent stages in *solo* mutants (S10 Fig), indicating that the inactivation of the alternative homolog conjunction during meiosis I occurs normally. However, THR depletion in *solo* mutants effectively prevented the disappearance of the SNM dot during exit from meiosis I (S10 Fig). Intense SNM dots were still present during meiosis II and during the early postmeiotic stages (S10 Fig). This perdurance of the alternative homolog conjunction presumably also explains the high frequency of chromosome bridges that was observed after THR depletion in *solo* mutants during meiosis II (Fig 3C).

### Biorientation of sister kinetochores during meiosis II does not depend on normal separase function

**Fig 4:**
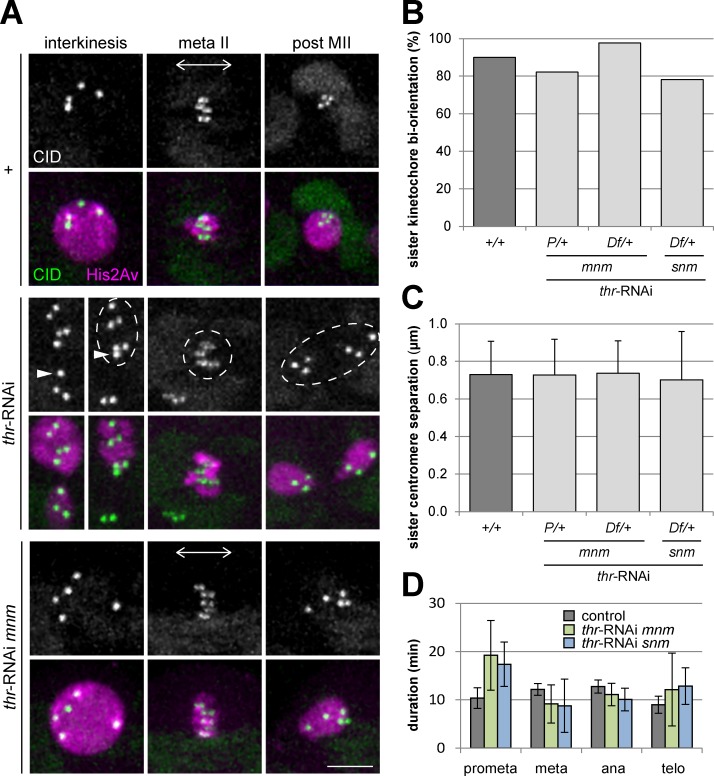
Sister kinetochore biorientation during meiosis II is not affected by THR depletion. (A) Still frames after time-lapse imaging of progression through meiosis II with testes expressing *cid-EGFP* (CID) and *His2Av-mRFP* (His2Av). Testes were from males without (+) or with spermatocyte-specific THR depletion by transgenic RNAi (*thr*-RNAi) as well as from *mnm* mutant males with identical spermatocyte-specific THR depletion (*thr*-RNAi *mnm*). During interkinesis before the onset of meiosis II, sister centromeres are not resolved and appear as a single Cid-EGFP dot. After sister kinetochore biorientation in the metaphase II spindle, sister centromeres are stretched apart along the spindle axis (double arrow). In control (+, top panel), sister centromeres are segregated far apart to opposite poles during exit from meiosis II; only one of the two postmeiotic nuclei with four centromeres is shown. However, after THR depletion (middle and bottom panel), sister centromere segregation to opposite poles during exit from meiosis II fails and each pair coalesces again into a single dot on the bridged (middle panel) or totally unpartitioned (bottom panel) chromatin mass present after meiosis II. Note that in the middle panel, a poorly visible fine chromatin bridge between two homologs is retracted at onset of the second meiotic division, and therefore one Cid-EGFP dot (arrowheads) joins the other dots in the upper cell (broken circle). Scale bar = 5 μm (B) Bars representing the percentage of single Cid-EGFP dots (n ≥ 43 Cid-EGFP dots) present before meiosis II that were separated along the spindle axis into two dots during metaphase II. The analyzed genotypes are indicated below the bars. *thr*-RNAi indicates spermatocyte-specific THR depletion. *mnm* and *snm* indicate loss-of-function mutations. *+*/*+* indicates the presence of two functional *thr* gene copies, while *P*/*+* and *Df*/*+* indicate heterozygosity for either a P-element disrupted *thr* gene or a *thr* deficiency, respectively. Precise genotype descriptions are given in S1 Table. (C) The distance between pairs of Cid-EGFP dots representing sister centromeres was measured during metaphase II. Bars represent average separation (n ≥ 34 pairs of Cid-EGFP dots), whiskers indicate s.d. The same genotypes and movies as in (B) were analyzed. (D) Bar diagram indicating average duration (± s.d.) of the indicated phases during meiosis II as revealed by time-lapse imaging of secondary spermatocyte cysts. The same genotypes and movies as in (B) were analyzed. The numbers of analyzed secondary spermatocytes were 5 (control), 8 (*thr*-RNAi in *mnm* mutants with only one functional *thr*^*+*^ gene copy), and 8 (*thr*-RNAi in *smn* mutants with only one functional *thr*^*+*^ gene copy).

The fact that separase is no longer required for chromosome separation during meiosis I in mutants with a non-functional alternative homolog conjunction system facilitates the analysis of a putative separase requirement for sister centromere individualization during progression through meiosis I. Since centromeric cohesin is thought to unite sister centromeres into a functional unit before meiosis I [12], destruction of centromeric cohesin by separase during meiosis I might enable sister centromere individualization and biorientation during meiosis II. After THR depletion in *mnm* mutants, univalents are randomly distributed into the two daughter cells. If separase function during meiosis I is required for sister centromere individualization, biorientation of these univalents in the meiosis II spindle is expected to fail and co-orientation of sister kinetochores as in meiosis I will also occur during meiosis II. To monitor sister centromere behavior we performed live imaging with spermatocytes expressing green fluorescent CID/Cenp-A as well as red fluorescent histone His2Av. Splitting of sister centromeres as well as sister kinetochore biorientation in meiosis II was clearly observed not only in control spermatocytes but also after THR depletion in *mnm* and *snm* mutants (Fig 4A and S7 and S9 Movies).

In fact, sister centromere splitting and biorientation could also be clearly detected after THR depletion in spermatocytes with functional homolog conjunction although meiosis II was often highly irregular as a result of the meiosis I chromosome separation failure (Fig 4A and S8 Movie). Importantly, sister centromere individualization and biorientation during meiosis II after THR depletion was detectable only transiently during metaphase II. During progression into metaphase II, the single centromere dots present in a secondary spermatocyte were split into a pair of dots along the spindle axis. However, the resolved sister kinetochores did not move apart towards opposite poles during anaphase II in the absence of separase function as a result of the failure in releasing sister chromatid cohesion.

While the sister chromatid separation failure indicated effective THR depletion, the presence of residual separase function still sufficient for sister centromere individualization during meiosis I cannot be ruled out definitively. To address this possibility, we carefully compared the effects of THR depletion in spermatocytes with either two or only one functional *thr* gene copy quantitatively. A reduction of the *thr* gene copy number is predicted to increase the efficiency of THR depletion and hence sister centromere splitting and kinetochore biorientation might be more compromised, if separase function during meiosis I is crucial for these processes. Therefore, we also performed THR depletion in spermatocytes heterozygous for either a P-element insertion within *thr* (*thr*^*k07805b*^) or a deficiency that deletes *thr* (*Df(2R)BSC338*). These THR depletion experiments were again performed in a background with mutations in *mnm* or *snm*, where meiosis II is not accompanied by spindle irregularities resulting from chromosome separation failure during meiosis I. We quantified the fraction of secondary spermatocytes that displayed centromere splitting during metaphase II (Fig 4B). Moreover, we also determined the inter-sister kinetochore distance during metaphase II (Fig 4C) because partial centromere individualization during meiosis I might result in a reduced inter-sister kinetochore distance during metaphase II. These measurements did not reveal a difference between metaphase II spermatocytes in controls and after THR depletion in spermatocytes with only one functional *thr* gene copy. Finally, by estimating the duration of the different phases (prometa-, meta-, ana-, telophase) in the time-lapse movies, we assessed whether the temporal dynamics of progression through meiosis II was affected by THR depletion in either *mnm* or *snm* mutants that had only one functional *thr*^*+*^ gene copy (Fig 4D). Compared to controls, the average time interval between nuclear envelope breakdown and anaphase onset was found to be slightly extended in the two THR depletion cases (28.4 ± 6.2 and 26. 1 ± 4.2 versus 22.5 ± 2.5 min; n = 8, 7 and 5, respectively) but not in a statistically significant manner. Similarly, prometaphase appeared to be extended in the two THR depletion cases (Fig 4D) although variablity in these two cases was considerable. Therefore, it is conceivable that separase makes a contribution to chromosome biorientation during meiosis II but it does not appear to be essential.

## Discussion

We demonstrate that the alternative system used for conjunction of homologous chromosomes before meiosis I that is used in Drosophila males instead of the canonical combination of chiasmata and sister chromatid cohesion, needs to be inactivated by separase during the transition from metaphase to anaphase for normal chromosome segregation during meiosis I. Moreover, we provide evidence that the individualization of sister kinetochores, which have been proposed to become united for co-orientation during meiosis I by centromeric cohesin, does not require separase function during exit from meiosis I. Our work also demonstrates that sister separation during meiosis II depends on separase.

As separase is required for development to the stages where male meiosis occurs, spermatocyte-specific depletion of separase complex subunits had to be developed for our analysis of separase function during male meiosis. In case of the THR subunit, transgenic RNAi expressed in early spermatocytes was found to be highly effective. Live imaging after THR depletion revealed a complete failure of homolog separation during meiosis I in all cells of all analyzed spermatocyte cysts. Similarly, cytological characterization of fixed testis squash preparations revealed chromosome bridges in late meiosis I figures (anaphase I, telophase I), consistent with the live imaging results. The 10% telophase I figures that did not display chromosome bridges in the fixed samples, at least in part, represent cases where the bridges did not persist long enough. We point out that chromosome bridges resulting from a failure of homolog separation during Drosophila male meiosis I are unlike those with continuous DNA throughout, resulting in mitosis after incomplete chromosome replication or in canonical meiosis after recombination defects. During Drosophila male meiosis I, homologs are thought to be conjoined by proteinaceous links that in exceptional cases might be severed eventually by spindle forces or other processes activated during exit from meiosis I. In principle, the few exceptional telophase I figures without chromosome bridges might also indicate residual THR function. To address the possibility of incomplete knockdown, we have carefully compared the effects of THR depletion in spermatocytes with either two or one functional copy of the endogenous *thr*^*+*^ gene. Since the effects of THR depletion were found to be entirely independent of the *thr*^*+*^ gene copy number, residual THR function does not appear to be present, although we cannot rule it out completely.

RNAi can have off-target effects. Several of our findings indicate that the consequences of THR depletion reported here do not reflect such off-target effects. Expression of the RNAi-resistant *UASt-thr*^*Rr*^ transgene resulted in significant although partial suppression of the THR depletion effects. Suppression simply as a result of Gal4 titration away from the *UAS-V20thr*^*shmiR9*^ transgene by the *UASt-thr*^*Rr*^ transgene can be ruled out, because experiments with two *UASt-thr*^*Rr*^ copies did not produce stronger suppression. We do not understand why *UASt-thr*^*Rr*^ expression does not result in complete suppression, but suspect problems caused by an inappropriately early temporal window of *bG*-mediated *UASt-thr*^*Rr*^ transgene expression, insufficient mRNA stability and translational control because of missing untranslated regions. Translational control is particularly pervasive in spermatocytes and is known to occur in case of Cyclin B and Twine/Cdc25 phosphatase, two well-studied cases of meiotic M phase regulators [52, 53]. We speculate that co-translation of all the separase complex subunits late during the four day spermatocyte growth phase might be required for the production of functional separase complexes for meiosis. *UASt-thr*^*Rr*^ transcripts are not present in late spermatocytes after expression using *bG*. Alternative GAL4 transgenes effectively driving expression in late spermatocytes do not exist.

The fact that SSE depletion in spermatocytes by deGradFP results in the same defects as THR depletion by RNAi provides further evidence against RNAi off-target effects. In case of SSE depletion by deGradFP, we were unable to achieve suppression by expression of an *UASp-Sse* transgene, presumably also for the reasons discussed above. These technical difficulties have precluded meaningful experiments addressing whether the effects of SSE depletion are suppressible by expression of an SSE variant predicted to be a catalytically inactive protease.

Our live imaging has revealed that the MNM-EGFP dot, which reflects XY chromosome conjunction, disappears very rapidly during the first 2–3 minutes of anaphase I in a THR-dependent manner. These observations and the corroborating analyses with anti-SNM on fixed samples strongly suggest that homolog separation during male meiosis I requires SSE protease activity, but they do not exclude a non-proteolytic role leading to MNM/SNM re-distribution throughout the cell that might not be detectable with our tools. A direct analysis of MNM/SNM protein levels during progression through meiosis I by immunoblotting would require the isolation of sufficient amounts of precisely staged meiotic cysts. The low abundance of meiosis I cysts has prevented such analyses so far. While the MNM-EGFP dot disappears during the cell cycle phase where separase is predicted to be active as a protease, a slightly earlier disappearance would have been expected. We suspect that proteolytic inactivation of the very high amounts of MNM/SNM present on the dot on the XY bivalent might be slower than the removal of the far less concentrated material from the autosome and that the XY bivalent therefore might be the last to separate during anaphase I.

Assuming that homolog separation during male meiosis I depends on protease activity of separase raises the question what the critical substrates might be. *Drosophila melanogaster* MNM and SNM contain sequences conforming to the consensus of separase cleavage sites [54], but they are poorly conserved within Drosophilid orthologs, and we have been unable to detect MNM and SNM cleavage during M phase after expression in mitotically proliferating cells (preliminary observations). As the molecular basis of homolog conjunction by MNM and SNM is far from being clear, it remains readily possible that they function together with additional unknown protein partners that might be cleaved by separase.

In principle, it appears conceivable that MNM and SNM co-operate with cohesin to bring about alternative homolog conjunction during male meiosis. The fact that SNM is a distant member of the SA/Stromalin family of cohesin subunits would appear to support this notion. Accordingly, separase might target α-kleisin as its critical substrate also during male meiosis I. Several observations argue against this. SNM as well as MNM do not co-localize with the SMC1 cohesin subunit [35] and therefore appear to function independent of cohesin. Moreover, the mitotic Rad21 α-kleisin does not appear to be involved during the meiotic divisions, at least in case of female meiosis [40]. The meiosis-specific α-kleisin family protein encoded in the Drosophila genome, C(2)M, is not required for male meiosis and does not function in a Rec8-like manner during female meiosis where it is required for normal synaptonemal complex formation but not sister chromatid cohesion [39, 55]. Genetically several genes have been identified that based on their mutant phenotype appear to provide a Rec8-like function during Drosophila meiosis [50, 51, 56–59]. Their protein products ORD, SOLO, and SUNN have no sequence similarity to α-kleisins. But they are mutually dependent on each other for their predominant localization around centromeres where they are co-occurring with SMC1 and SMC3. All evidence therefore suggests that these proteins might function in a Drosophila-specific variant cohesin complex providing sister chromatid cohesion during meiosis. While it is readily possible that one of these proteins is a separase substrate during meiotic divisions, this would not explain how separase brings about homolog separation during male meiosis I for reasons also provided by our experiments. We demonstrate that homolog conjunction by MNM and SNM during male meiosis does not depend on *solo* function, consistent with previous work [50]. This conclusion is suggested by our observations that THR depletion in *solo* mutants results in chromosome bridges during meiosis I which are no longer observed when THR is depleted in *solo mnm* or *solo snm* double mutants. In the absence of *solo* function, therefore, MNM/SNM establish chromosome conjunction that results in chromosome bridges during meiosis I in the absence of separase function. At present, a critical target that needs to be cleaved by separase for homolog separation during male meiosis I is not known. Similarly, the critical separase target for sister chromatid separation during meiosis II is also unknown.

The possibility to by-pass the separase requirement for chromosome separation during meiosis I in Drosophila males by mutational inactivation of the alternative homolog conjunction system, in combination with our ability to monitor progression through both meiotic divisions by time-lapse imaging, has also allowed us to address the role of separase for sister centromere individualization during exit from meiosis I. A series of extremely elegant experiments in fission yeast has provided strong evidence suggesting that the co-orientation of sister centromeres that is established specifically during meiosis I for biorientation of bivalents in meiosis I spindles depends on the presence of centromeric sister chromatid cohesion mediated by meiotic Rec8 cohesin complexes [9, 26, 29, 30]. Centromeric cohesion that keeps sister centromeres in close proximity might result in the assembly of a single kinetochore on each homolog at the onset of meiosis I. Importantly, to allow sister kinetochore biorientation within meiosis II spindles, centromeric cohesion would have to be resolved at some stage after co-orientation during meiosis I has been achieved. Our observations suggest that separase might not be required for sister centromere individualization during Drosophila male meiosis. We demonstrate that sister kinetochore biorientation is successful during meiosis II after THR depletion, while sister chromatid separation is completely abolished. Separase might be dispensable for the removal of centromeric cohesin during meiosis I because of an alternative cohesin removal mechanism. In principle, Wapl can open cohesin rings without separase although only when the rings have not yet been locked by SMC3 acetylation [60–64]. Alternatively, sister centromere co-orientation in Drosophila male meiosis might not involve centromeric cohesin. Finally, we acknowledge that our evidence cannot definitely rule out the possibility that some residual THR escaping depletion might still be sufficient for normal sister kinetochore biorientation but not for sister chromatid separation during meiosis II. Moreover, we also point out that our light microscopic analyses cannot resolve the aspects of sister centromere individualization during Drosophila male meiosis I that have been observed by serial sectioning and electron microscopic analysis [7]. At the ultrastructural level, a hemispherical kinetochore, where the two sister kinetochores cannot be resolved, is detected in early prometaphase I spermatocytes. By anaphase I, however, two closely associated but clearly distinct sister kinetochores in a side-by-side configuration were usually observed. For lack of spatial resolution, we cannot exclude that extent or dynamics of this sister kinetochore resolution process during meiosis I is abnormal after THR depletion. Similarly, our data can also not exclude that a possible separase contribution to sister kinetochore biorientation during meiosis II might eventually be compensated during prometaphase II in THR depleted spermatocytes by spindle forces for example. Our limited data on temporal dynamics of chromosome congression during meiosis II after THR depletion in *mnm* and *snm* mutants is consistent with the notion that separase makes some contribution to efficient chromosome biorientation during meiosis II but cannot prove it. The analysis of the role of separase for sister kinetochore biorientation during meiosis certainly deserves further attention, including studies in other organisms.

## Materials and Methods

### Drosophila lines

The following lines with previously characterized mutations or transgenes were used: *Sse*^*13m*^ and *Df(3L)SseA* [42], *thr*^*k07805b*^ [65], *Df(2R)BSC338* [66], *P{ry+*, *hsp70-mnm-EGFP}*, *mnm*^*Z3-3298*^, *mnm*^*Z3-5578*^, *snm*^*Z3-0317*^, and *snm*^*Z3-2138*^ [35], *solo*^*Z2-0198*^ and *solo*^*Z2-0338*^ (Yan et al., 2010), *tef*^*Z2-4169*^ and *tef*^*Z2-3455*^ [67], *P{w*^*+*^, *bamP-GAL4-VP16}III* [68], *P{w*^*+*^, *His2Av-mRFP}II*.*2* and *P{w*^*+*^, *gcid-EGFP-cid}II*.*1* [69], *P{w*^*+*^, *pUbi-EGFP-alphaTub84B}II* [70], and *P{w*^*+*^, *pUbi-EYFP-asl}* [71].

Lines for transgenic RNA interference, *UAS-V20thr*^*shmiR9*^, *UAS-V20thr*^*shmiR10*^ and *UAS-W20thr*^*shmiR45*^, were generated by integrating the pVALIUM20 and pWALIUM20 constructs (see below) into the attP2 landing site. For production of *P{w*^*+*^, *UASt-thr*^*Rr*^*}attP40*, allowing expression of a *thr* cDNA with silent mutations in the regions targeted by *thr*^*shmiR9*^, *thr*^*shmiR10*^ and *thr*^*shmiR45*^, a pUASt-attB construct (see below) was integrated into the attP40 landing site. For *pim*-RNAi, we used *y w*^*1118*^*; P{w*^*+*^, *KK106514}VIE-260B* (v100534). *PBac{3xP3-ECFP*, *gEGFP-Sse}III*.*1* was generated by germline transformation with a PiggyBac construct. *P{w*^*+*^, *bamP-NSlmb-vhh-GFP4}II*.*1* was isolated after P-element-mediated germline transformation with a pCaSpeR4 construct.

For all experiments, flies were cultured at 25°C. Detailed genotypes of the flies analyzed are provided in the supplemental material (S1 Table).

### Plasmids

For the production of transgenic lines allowing GAL4-dependent expression of short hairpin microRNAs (shmiRs), we generated constructs using the vectors pVALIUM20 and pWALIUM20 [72]. Inserts were generated by annealing the following oligonucleotides: 5'-ctagcagt-CCCTTGGAAGCTACAAGTCAA-tagttatattcaagcata-TTGACTTGTAGCTTCCAAGGG-gcg-3' and 5'-aattcgc-CCCTTGGAAGCTACAAGTCAA-tatgcttgaatataacta-TTGACTTGTAGCTTCCAAGGG-actg-3' for *thr*^*shmir9*^, 5'-ctagcagt-AACGCTTCTAGTTCAACTAAA-tagttatattcaagcata-TTTAGTTGAACTAGAAGCGTT-gcg-3' and 5'-aattcgc-AACGCTTCTAGTTCAACTAAA-tatgcttgaatataacta-TTTAGTTGAACTAGAAGCGTT-actg-3') for *thr*^*shmir10*^, 5’-ctagcagt-AAGAAGTAGATCATTCTTCAA-tagttatattcaagcata-TTGAAGAATGATCTACTTCTT-gcg-3’ and 5’-aattcgc-AAGAAGTAGATCATTCTTCAA-tatgcttgaatataacta-TTGAAGAATGATCTACTTCTT-actg-3’ for *thr*^*shmir45*^. Capital letters indicate the regions corresponding to sequences within the *thr* coding sequence.

For the production of a transgenic line allowing expression of *EGFP-Sse* under control of the *Sse* cis-regulatory region, we generated *pBac{3xP3-ECFP-gEGFP-Sse}* using a modified version of the previously described *gSse* transgene construct [42]. During construction, an *Sse* cDNA fragment replacing the *Sse* genomic region containing the first two introns was introduced as well as the EGFP coding sequence fused at the N-terminus.

For the construction of pCaSpeR4-bamP-NSlmb-vhh-GFP4, the fragment coding for NSlmb-vhh-GFP4 was amplified from pUASt-NSlmb-vhh-GFP4 [49] using primers SCH1 (5’-GACTACCGGTATGATGAAAATGGAGACTGAC-3’) and SCH2 (5’-GACTGCGGCCGCTTAGCTGGAGACGGTGAC-3’). After digestion with AgeI and NotI, the insert was used to replace the GAL4-VP16 coding region released by the same restriction enzymes in pCaSpeR4-bamP-GAL4-VP16 [68].

A modified *thr* cDNA was inserted into pUASt-attB [73] for the production of pUASt-attB-thr^Rr^. The *thr* cDNA [48] was modified by replacing the region containing shmiR target sequences with a synthetic variant (GenScript, Piscataway, NJ 08854, USA).

### Fertility tests

The fertility of 5–10 single males per genotype was assayed in parallel. Each single male was allowed to mate with three *w* virgin females for two days. Flies were transferred into a fresh vial and discarded after two more days. Flies eclosing from this vial were counted.

### Testis preparations

Testis squash preparations were done as described [74]. For immunolabeling, mouse monoclonal anti-α-tubulin DM1A (Sigma) was used at 1:10’000 and affinity-purified rabbit polyclonal anti-SNM [35] at 1:250. Secondary antibodies were Alexa488- or Alexa568-conjugated goat antibodies against mouse or rabbit IgG diluted 1:1000.

### Fluorescence in situ hybridization (FISH)

Dissection of testis, fixation with 4% PFA, permeabilization with PBST-DOC and anti- α-tubulin staining were done as described (protocol 3.2.2, steps 1–14) [74]. Cy5-conjugated goat anti-mouse IgG diluted 1:1000 was used as secondary antibody. Ethanol incubations and dehydration with a formamide series were also done as described (immuno-FISH protocol 3.2, steps 10–26) [75]. An oligonucleotide (5'-TTTTCCAAATTTCGGTCATCAAATAATCAT-3') with Atto-565 on 5’ and 3’ end (Integrated DNA Technologies, B-3001 Leuven Belgium) was used for detection of the X-specific 359 bp repeats at a concentration of ≈ 1 ng/μl in hybridization Buffer. An oligonucleotide (5'-AATACAATACAATACAATACAATACAATAC-3') with Alexa-488 fluorophore at the 3’ end (Sigma-Aldrich, 8107 Buchs, Switzerland) was used for detection of the Y-specific AATAC repeats at a concentration of ≈ 2 ng/μl in hybridization buffer. The denaturation step was performed at 98°C for 6 min, and hybridization over night at 16°C. Slides were washed twice in 50% formamide, 2x SSCT at 16°C for 1 hour each. Thereafter, additional washes were performed at room temperature in 25% formamide, 2x SSCT for 10 min and three times in 2x SSCT for 10 min each. DNA was stained with Hoechst 33258 (1 μg/ml) for 10 min and slides were washed twice in PBS for 5 min. Slides were mounted in 70% Glycerol, 50 mM Tris-HCl pH 8.5, 10 mg/ml propyl gallate, 0.5 mg/ml phenylendiamine.

### Microscopy and image analysis

Image stacks with 250 nm spacing between focal planes were acquired with a 63×/1.4 oil immersion objective on a Zeiss Cell Observer HS microscope. If not stated differently, the images displayed in the figures represent maximum intensity projections. The data used for statistical analyses of a particular genotype was obtained from multiple slides and each slide was prepared with about 14 dissected testes.

### Time-lapse imaging

Testes from pupal or adult males were dissected in Schneider’s Drosophila Medium (Invitrogen, #21720), 10% fetal bovine serum (Invitrogen), 1% penicillin/streptomycin (Invitrogen, #15140). The dissected testes were transferred into 40 μl of medium in a 35 mm glass bottom dish (MatTek Corporation, #P35G-1.5-14-C), and opened with fine tungsten needles to release the cysts. To reduce movements within the sample, methylcellulose (Sigma) was added. A wet filter paper was placed inside along the dish wall before sealing the lid with parafilm. Time-lapse imaging was performed with a spinning disc confocal microscope (Visitron) with a 60×/1.4 oil immersion objective. Image stacks with 24–45 focal planes spaced by 0.5–1 μm were acquired with a time interval of 30–60 sec. Precise numbers are specified in the legends for each supplementary movie. The distance between sister centromeres during metaphase II was measured in 3D using Imaris software (Bitplane). To exclude that the measurements actually represent early anaphase time points, the last three time points before anaphase onset were excluded from consideration.

## Supporting Information

S1 Table Genotypes and meiotic phenotypes. Full genotypes of the males used for testis dissection and analysis are presented along with data concerning fertility and chromosome bridging during telophase I and telophase II.
(PDF)

S1 Fig Expression of the RNAi-resistant *thr*^*Rr*^ transgene suppresses the effect of *thr*-RNAi. Squash preparations were labeled with anti-tubulin (tubulin) and a DNA stain (DNA). Testes were isolated from males without (+, left row) or with spermatocyte-specific THR depletion by transgenic RNAi (*thr*-RNAi, middle row), as well as from males in which spermatocyte-specific THR depletion was combined with expression of a *thr* transgene (*UASt-thr*^*Rr*^) predicted to be RNAi-resistant as a result of silent mutations (*thr*-RNAi *+* rescue, right row). Precise genotype descriptions are given in S1 Table. The comparison of telophase I cysts reveals that *thr*-RNAi induces frequent and strong chromosome bridges which are strongly suppressed by the RNAi-resistant transgene. Scale bar = 10 μm.
(TIF)

S2 Fig SSE depletion by deGradFP results in chromosome bridges during telophase I and II. Squash preparations were labeled with anti-tubulin (tubulin) and a DNA stain (DNA). Testes were isolated from males without (+, upper panel) or with spermatocyte-specific SSE depletion by deGradFP (*Sse*-deGrad, middle panel), as well as from males with spermatocyte-specific PIM depletion by RNAi (*pim*-RNAi, lower panel). Precise genotype descriptions are given in S1 Table. The comparison of the cysts at the indicated meiotic stages reveals that SSE depletion induces chromosome bridges during meiosis I and II. Scale bar = 10 μm.
(TIF)

S3 Fig SNM persists throughout meiosis II upon THR depletion Testes were isolated from *mnm-EGFP* males with spermatocyte-specific THR depletion (*thr*-RNAi). Squash preparations were labeled with anti-SNM (SNM), anti-tubulin (tubulin) and a DNA stain (DNA). Single spermatocytes at the indicated stages during meiosis II (scale bar = 5 μm) and part of a postmeiotic cyst (scale bar = 10 μm) are displayed.
(TIF)

S4 Fig SSE or PIM depletion causes failure of chromosomal SNM release and formation of chromosome bridges in telophase I and II. Testes were isolated from males without (+, upper panel) or with spermatocyte-specific SSE depletion by deGradFP (*Sse*-deGrad, middle panel), as well as from males with spermatocytes-specific PIM depletion by RNAi (*pim*-RNAi, lower panel). Squash preparations were labeled with anti-SNM (SNM), anti-tubulin (tubulin) and a DNA stain (DNA). Single spermatocytes at the indicated meiotic stages are displayed. Scale bar = 5 μm.
(TIF)

S5 Fig *mnm* and *snm* are required for induction of telophase I chromosome bridges by THR or SSE depletion. Squash preparations were labeled with anti-tubulin (tubulin) and a DNA stain (DNA). Testes were isolated from males with the indicated genotypes. + indicates absence of THR and SSE depletion as well as absence of *mnm* and *snm* mutations. *mnm* and *snm* indicate loss-of-function mutations. *thr*-RNAi and *Sse*-deGrad indicate spermatocyte-specific depletion with transgenic RNAi and deGradFP, respectively. Precise genotype descriptions are given in S1 Table. THR and SSE depletion do not induce bridges during meiosis I when the alternative homolog conjunction system does not function as a result of mutations in *mnm* or *snm*. Scale bar = 10 μm.
(TIF)

S6 Fig THR and SSE depletion inhibit X-Y separation during meiosis I. Squash preparations were labeled with anti-tubulin (tubulin) and a DNA stain (DNA). In addition, FISH with a red fluorescent X chromosome probe (X) and a green fluorescent Y chromosome probe (Y) was performed. As the X probe hybridizes close to the centromere (heterochromatic region h31), a single dot representing both sister chromatids is usually observed during meiosis I. In contrast, the Y probe which hybridizes to a region within the long arm (h6) usually generates two dots during meiosis I, one for each sister chromatid. The displayed single spermatocytes during telophase I were from males without (+) or with spermatocyte-specific SSE depletion by deGradFP (*Sse*-deGrad). In addition, *mnm* mutant males with identical spermatocyte-specific SSE depletion are displayed as well (*Sse*-deGrad *mnm*). In control (+), chromosome bridges were absent (without bridge) and X and Y were segregated into opposite daughter nuclei (XX<->YY). After *Sse*-deGrad, only very few telophase I figures were apparently normal, while the large majority had a chromosome bridge (with bridge) or complete separation failure which was also scored as bridged. In some cases, X and Y were on opposite sides of these bridges (XX<->YY). However, more frequently X and Y were not segregated apart (XXYY) and signals were observed at various positions along the bridge. Loss of *mnm* function was found to suppresses chromosome bridge induction by *Sse*-deGrad and was associated with random segregation of X and Y chromosomes into either the same daughter nucleus (XXYY) or into opposite daughter nuclei (XX<->YY). The frequency of these phenotypes observed in the different genotypes is given at the bottom in a table that includes data from THR depletion by transgenic RNAi.
(TIF)

S7 Fig THR depletion specifically inhibits homolog separation during meiosis I. Live imaging performed with testes from *cid-EGFP His2Av-mRFP* males which have green fluorescent centromeres and red fluorescent chromatin. In addition, spermatocyte-specific THR depletion was either absent (*+*) or present (*thr*-RNAi). Still frames are presented from a representative control spermatocyte and three *thr*-RNAi spermatocytes during progression through meiosis I. The still frames were oriented so that the spindle axis is horizontal. Moreover, still frames where centromere number and positions were most clearly resolved during a particular meiotic stage were selected from a given movie. The second still frame always illustrates very late metaphase I. The first still frames illustrating prometaphase I were between 11 and 15 minutes before the late metaphase I frame. The third still frames (chromosome decondensation) were between five and six minutes after the late metaphase I frame. The fourth still frames (post MI) were between 14 and 20 minutes after the late metaphase I frame. In case of the three *thr*-RNAi spermatocytes, the fifth still frames were between 47 and 57 minutes after the late metaphase I frame, revealing bridging in two cases as well as complete separation failure in one case (bottom). While *thr*-RNAi prevents homolog separation, it interferes neither with the temporal dynamics of meiosis I progression nor with alignment of the bivalents into the metaphase I plate, indicating that spindles and kinetochores are functional. Scale bar = 5 μm.
(TIF)

S8 Fig *solo* function is required for *thr*-RNAi mediated induction of chromosome bridges in *snm* mutants during telophase II. Squash preparations were labeled with anti-tubulin (tubulin) and a DNA stain (DNA). Testes were isolated from males with the indicated genotypes. *thr*-RNAi indicates spermatocyte-specific THR depletion by transgenic RNAi. *solo* and *snm* indicate loss-of-function mutations. Precise genotype descriptions are given in S1 Table. The comparison of the cysts at the indicated meiotic stages reveals that the chromosome bridges during telophase II which are observed after THR depletion in *snm* mutants, are no longer detectable when spermatocytes also lack *solo* function. Scale bar = 10 μm.
(TIF)

S9 Fig Alternative homolog conjunction is functional in *solo* mutants and results in telophase I bridges after THR depletion in these mutants. Squash preparations were labeled with anti-tubulin (tubulin) and a DNA stain (DNA). Testes were isolated from males with the indicated genotypes. *thr*-RNAi indicates spermatocyte-specific THR depletion by transgenic RNAi. *solo*, *mnm* and *snm* indicate loss-of-function mutations. Precise genotype descriptions are given in S1 Table. The comparison of the cysts at the indicated meiotic stages reveals that the chromosome bridges during telophase I, which are observed after THR depletion in *solo* mutants, are no longer detectable in spermatocytes which are unable to perform homolog conjunction due to lack of *mnm* or *snm* function. Scale bar = 10 μm.
(TIF)

S10 Fig THR depletion in *solo* mutant causes failure of chromosomal SNM release and formation of chromosome bridges in telophase I and II. Testes were isolated from *solo* mutant males without (*solo*) or with spermatocyte-specific THR depletion (*thr*-RNAi *solo*), as well as from males without spermatocyte-specific THR depletion (+). Squash preparations were labeled with anti-SNM (SNM), anti-tubulin (tubulin) and a DNA stain (DNA). Single spermatocytes at the indicated stages (A, scale bar = 5 μm) and part of a postmeiotic cyst (B, scale bar = 10 μm) are displayed.
(TIF)

S1 Movie Absence of chromosome bridges in telophase I and II during normal meiosis. Time-lapse analysis of progression through the first and second meiotic divisions in spermatocytes expressing *EGFP-tubulin* and *His2Av-mRFP*. Both meiotic divisions are symmetrical and no chromosome bridges occur. Precise genotype description is given in S1 Table. Image stacks with 24 focal planes spaced by 1 μm were acquired with a time interval of 1 min. Scale bar = 10 μm.
(AVI)

S2 Movie THR depletion by transgenic RNAi results in chromosome bridges in telophase I and II. Time-lapse analysis of progression through the first and second meiotic divisions in spermatocytes expressing *EGFP-tubulin* and *His2Av-mRFP*. Moreover, spermatocyte-specific THR depletion was induced by transgenic RNAi (*thr*-RNAi). Chromosome separation during anaphase I and II did not succeed in THR depleted cells resulting in abnormal telophase with unseparated chromosomes squeezed apart by the contractile furrow into two daughter cells or as a single mass into one daughter cell. As a consequence of the meiosis I segregation failure, spindles in meiosis II are often irregular due to chromatin in the division plane. Precise genotype description is given in S1 Table. Image stacks with 24 focal planes spaced by 1 μm were acquired with a time interval of 1 min. Scale bar = 10 μm.
(AVI)

S3 Movie Rapid chromosomal MNM release and homolog separation during normal meiosis I. Time-lapse analysis of progression through the first meiotic division in spermatocytes expressing *mnm-EGFP* and *His2Av-mRFP*. The MNM-EGFP spot disappears during early anaphase I within 3 minutes. Precise genotype description is given in S1 Table. Image stacks with 30 focal planes spaced by 0.5 μm were acquired with a time interval of 30 sec. Scale bar = 5 μm.
(AVI)

S4 Movie THR depletion causes failure of chromosomal MNM release and homolog separation during meiosis I. Time-lapse analysis of progression through the first meiotic division in spermatocytes expressing *mnm-EGFP* and *His2Av-mRFP*. Moreover, spermatocyte-specific THR depletion was induced by transgenic RNAi (*thr*-RNAi), resulting in persistence of the MNM-EGFP spot and chromosome separation failure. Precise genotype description is given in S1 Table. Image stacks with 30 focal planes spaced by 0.5 μm were acquired with a time interval of 30 sec. Scale bar = 5 μm.
(AVI)

S5 Movie Biorientation and separation of bivalents during normal meiosis I. Time-lapse analysis of progression through the first meiotic division in spermatocytes expressing *cid-EGFP* and *His2Av-mRFP*. The movie starts at the nuclear envelope breakdown. In prometaphase I, up to 4 DNA masses, each with two green centromere dots, are recognizable. Bivalents align into a metaphase I plate and homologous chromosomes segregate into the daughter cells. Precise genotype description is given in S1 Table. Image stacks with 30 focal planes spaced by 1 μm were acquired with a time interval of 1 min. Scale bar = 5 μm.
(AVI)

S6 Movie Normal biorientation of bivalents followed by separation failure during meiosis I after THR depletion. Time-lapse analysis of progression through the first meiotic division in spermatocytes expressing *cid-EGFP* and *His2Av-mRFP*. Moreover, spermatocyte-specific THR depletion was induced by transgenic RNAi (*thr*-RNAi). The movie starts at the nuclear envelope breakdown. In prometaphase I, up to 4 DNA masses, each with two green centromere dots, are recognizable. Bivalents align into a metaphase I plate indicating that spindles and kinetochores are functional. However, homologous chromosomes fail to separate during anaphase I. Precise genotype description is given in S1 Table. Image stacks with 30 focal planes spaced by 1 μm were acquired with a time interval of 1 min. Scale bar = 5 μm.
(AVI)

S7 Movie Sister kinetochore biorientation and segregation during normal meiosis II. Time-lapse analysis of progression through the second meiotic division in spermatocytes expressing *cid-EGFP* and *His2Av-mRFP*. During interkinesis before the onset of meiosis II, sister centromeres are not resolved and appear as a single Cid-EGFP dot. After sister kinetochore biorientation in the metaphase II spindle, sister centromeres are stretched apart along the spindle axis and segregated to opposite poles during anaphase II. Precise genotype description is given in S1 Table. Image stacks with 20 focal planes spaced by 1 μm were acquired with a time interval of 1 min. Scale bar = 5 μm.
(AVI)

S8 Movie Normal sister kinetochore biorientation followed by segregation failure during meiosis II after THR depletion. Time-lapse analysis of progression through the second meiotic division in spermatocytes expressing *cid-EGFP* and *His2Av-mRFP*. Moreover, spermatocyte-specific THR depletion was induced by transgenic RNAi (*thr*-RNAi). During interkinesis before the onset of meiosis II, sister centromeres are not resolved and appear as a single Cid-EGFP dot. After normal sister kinetochore biorientation in the metaphase II spindle, sister centromeres are stretched apart along the spindle axis. However, sister centromere segregation to opposite poles during exit from meiosis II fails and each pair of sister centromeres coalesces again into a single dot on the bridged chromatin mass present after meiosis II. A poorly visible fine chromatin bridge between two homologs is retracted at onset of the second meiotic division, and therefore one Cid-EGFP dot joins the other dots in the upper cell. Precise genotype description is given in S1 Table. Image stacks with 15 focal planes spaced by 1 μm were acquired with a time interval of 1 min. Scale bar = 5 μm.
(AVI)

S9 Movie Normal sister kinetochore biorientation followed by segregation failure during meiosis II after THR depletion in *mnm* mutants. Time-lapse analysis of progression through the second meiotic division in *mnm* mutant spermatocytes expressing *cid-EGFP* and *His2Av-mRFP*. Moreover, spermatocyte-specific THR depletion was induced by transgenic RNAi (*thr*-RNAi). During interkinesis before the onset of meiosis II, sister centromeres are not resolved and appear as a single Cid-EGFP dot. After normal sister kinetochore biorientation in the metaphase II spindle, sister centromeres are stretched apart along the spindle axis. However, sister centromere segregation to opposite poles during exit from meiosis II fails and each pair of centromeres coalesces again into a single dot on the unpartitioned chromatin mass present after meiosis II. Precise genotype description is given in S1 Table. Image stacks with 12 focal planes spaced by 1 μm were acquired with a time interval of 1 min. Scale bar = 5 μm.
(AVI)
