# Supplementary material for: Separase Is Required for Homolog and Sister Disjunction during Drosophila melanogaster Male Meiosis, but Not for Biorientation of Sister Centromeres
Source: PLoS Genet. 2016 Apr 27;12(4):e1005996. doi: 10.1371/journal.pgen.1005996 (PMC4847790; doi:10.1371/journal.pgen.1005996)
Supplement: S1 Table — Full genotypes of the males used for testis dissection and analysis are presented along with data concerning fertility and chromosome bridging during telophase I and telophase II. (PDF) [file pgen.1005996.s001.pdf]

S1 Table

Squash preparations

abbreviation	full genotype [a)]	fertility [b)]	telophase I cells with bridges	telophase II cysts with bridges [f)]
+ (RNAi)	w*/Y;; P{w+, bamP-GAL4-VP16}/+	100% (n = 50)	0% (n = 262)	0% (n = 17)
thr -RNAi	w*/Y;; P{w+, bamP-GAL4-VP16}/P{v+, UAS-V20thr^{shmiR9} }attP2	0% (n = 40)	86% [c)] (n = 145)	100% (n = 10)
thr -RNAi<br>+ rescue	w*/Y; P{w+, UASt-thr^{Rr} }attP40/+;<br>P{w+, bamP-GAL4-VP16}/P{v+, UAS-V20thr^{shmiR9} }attP2	0% (n = 10)	24% (n = 391)	100% (n = 10)
	w* P{w+, pUbi-EYFP-asl}/Y;;<br>P{w+, bamP-GAL4-VP16}/P{v+, UAS-V20thr^{shmiR10} }attP2	0% (n = 5)	n.d.	n.d.
	w* P{w+, pUbi-EYFP-asl}/Y;;<br>P{w+, bamP-GAL4-VP16}/P{w+, UAS-W20thr^{shmiR45} }attP2	0% (n = 10)	n.d.	n.d.
pim -RNAi	w*/Y; +/P{w+, KK106514}VIE-260B; P{w+, bamP-GAL4-VP16}/+	3% (n = 19)	56% (n = 164)	100% (n = 18)
+ (deGrad)	w*/Y;; Df(3L)SseA st e/PBac{3xP3-ECFP, gEGFP-Sse}III.1 Sse^{13m}	100% (n = 20)	1% (n = 371)	5% (n = 22)
Sse -deGrad	w*/Y; P{w+, bamP-NSlmb-vhh-GFP4}II.1/+;<br>Df(3L)SseA st e/PBac{3xP3-ECFP, gEGFP-Sse}III.1 Sse^{13m}	0% (n = 20)	90% [d)] (n = 540)	100% (n = 23)
mnm	+/Y; bw/+; mnm$^{Z3-5578}$ /mnm$^{Z3-3298}$	36% (n = 10)	2% [e)] (n = 165)	10% (n = 10)
snm	w*/Y; bw/+; snm$^{Z3-2138}$ /snm$^{Z3-0317}$	36% (n = 10)	10% [e)] (n = 82)	17% (n = 6)
tef	+/Y; cn tef$^{Z2-4169}$ bw/cn tef$^{Z2-3455}$ bw	64% (n = 15)	1% (n = 186) [e)]	0% (n = 5)
thr -RNAi mnm	w*/Y;; P{w+, bamP-GAL4-VP16} mnm$^{Z3-3298}$ /<br>P{v+, UAS-V20thr^{shmiR9} }attP2 mnm$^{Z3-5578}$	25% (n = 20)	0% (n = 97)	100% (n = 5)
thr -RNAi snm	w*/Y;; P{w+, bamP-GAL4-VP16} snm$^{Z3-0317}$ /<br>snm$^{Z3-2138}$ P{v+, UAS-V20thr^{shmiR9} }attP2	2% (n = 20)	0% (n = 148)	100% (n = 5)
Sse -deGrad mnm	w*/Y; P{w+, bamP-NSlmb-vhh-GFP4}II.1/+; Df(3L)SseA mnm$^{Z3-5578}$ /<br>PBac{3xP3-ECFP, gEGFP-Sse}III.1 Sse^{13m} mnm$^{Z3-3298}$	0% (n = 10)	0% (n = 158)	100% (n = 13)
Sse -deGrad snm	w*/Y; P{w+, bamP-NSlmb-vhh-GFP4}II.1/+; snm$^{Z3-2138}$ Df(3L)SseA/<br>PBac{3xP3-ECFP, gEGFP-Sse}III.1 snm$^{Z3-0317}$ Sse^{13m}	0% (n = 10)	1% [e)] (n = 112)	100% (n = 10)
solo	+/Y; solo$^{Z2-0198}$ cn bw/solo$^{Z2-0338}$ cn bw	32% (n = 40)	1% [e)] (n = 106)	17% (n = 6)
thr-RNAi solo	w*/Y; solo$^{Z2-0198}$ cn bw/solo$^{Z2-0338}$ cn bw ;<br>P{w+, bamP-GAL4-VP16}/P{v+, UAS-V20thr^{shmiR9} }attP2	0% (n = 25)	94% (n = 127)	100% (n = 12)
mnm solo	w*/Y; solo$^{Z2-0198}$ cn bw/solo$^{Z2-0338}$ cn bw; mnm$^{Z3-3298}$ /mnm$^{Z3-5578}$	22% (n = 20)	0% (n = 79)	0% (n = 16)
snm solo	w*/Y; solo$^{Z2-0198}$ cn bw/solo$^{Z2-0338}$ cn bw; snm$^{Z3-0317}$ /snm$^{Z3-2138}$	16% (n = 6)	0% (n = 24)	0% (n = 4)
thr -RNAi mnm solo	w*/Y; solo$^{Z2-0198}$ /solo$^{Z2-0338}$; P{w+, bamP-GAL4-VP16} mnm$^{Z3-3298}$ /<br>P{v+, UAS-V20thr^{shmiR9} }attP2 mnm$^{Z3-5578}$	0% (n = 16)	0% (n = 173)	0% (n = 9)
thr -RNAi snm solo	w*/Y; solo$^{Z2-0198}$ /solo$^{Z2-0338}$; P{w+, bamP-GAL4-VP16} snm$^{Z3-0317}$ /<br>snm$^{Z3-2138}$ P{v+, UAS-V20thr^{shmiR9} }attP2	1% (n = 19)	0% (n = 104)	0% (n = 9)
thr -RNAi tef	w*/Y; cn tef$^{Z2-4169}$ bw/cn tef$^{Z2-3455}$ bw;<br>P{w+, bamP-GAL4-VP16}/P{v+, UAS-V20thr^{shmiR9} }attP2	0% (n = 10)	25% (n = 353)	100% (n = 25)
MNM-EGFP	w*/Y; P{ry+, hsp70-mnm-EGFP}/+ ; P{w+, bamP-GAL4-VP16}/+	n.d.	n.d.	n.d.
MNM-EGFP thr-RNAi	w*/Y; P{ry+, hsp70-mnm-EGFP}/+ ;<br>P{w+, bamP-GAL4-VP16}/P{v+, UAS-V20thr^{shmiR9} }attP2	n.d.	n.d.	n.d.

a) maternal/paternal chromosome

b) n = number of single males analyzed in total, 100% corresponds to 205 ± 25 F1 progeny per single male on average

c) 19% had a single mass of undivided chromosomes rather than a bi-lobed mass

d) 8% had a single mass of undivided chromosomes rather than a bi-lobed mass

e) telophase I chromosome bridges presumably reflecting lagging chromosomes after premature separation rather than separation failure

f) While chromosome separation abnormalities were observed in the large majority of secondary spermatocytes within the same cyst in case of genotypes scoring 100%, only few secondary spermatocytes were affected in the other genotypes with more than 0% presumably reflecting lagging chromosomes after premature separation rather than separation failure.

Time-lapse imaging

abbreviation	full genotype
MNM-EGFP His2Av-mRFP	w*/Y; P{w+, His2Av-mRFP}II.2/P{ry+, hsp70-mnm-EGFP}
MNM-EGFP His2Av-mRFP<br>thr-RNAi	w*/Y; P{w+, His2Av-mRFP}II.2/P{ry+, hsp70-mnm-EGFP},<br>P{w+, bamP-GAL4-VP16}/P{v+, UAS-V20thr^{shmiR9} }attP2
CID-EGFP His2Av-mRFP	w*/Y; {w+, gCid-EGFP-Cid}II.1 P{w+, His2Av-mRFP}II.2/+,<br>P{w+, bamP-GAL4-VP16}/+
CID-EGFP His2Av-mRFP<br>thr-RNAi	w*/Y; {w+, gCid-EGFP-Cid}II.1 P{w+, His2Av-mRFP}II.2/+,<br>P{w+, bamP-GAL4-VP16}/P{v+, UAS-V20thr^{shmiR9} }attP2
CID-EGFP His2Av-mRFP<br>thr-RNAi mnm	w*/Y; {w+, gCid-EGFP-Cid}II.1 P{w+, His2Av-mRFP}II.2+,<br>P{w+, bamP-GAL4-VP16} mnm$^{Z3-3298}$ /<br>P{v+, UAS-V20thr^{shmiR9} }attP2 mnm$^{Z3-5578}$
CID-EGFP His2Av-mRFP<br>P/+ thr-RNAi mnm	w*/Y; {w+, gCid-EGFP-Cid}II.1 P{w+, His2Av-mRFP}II.2/thr^{k07805b} ;<br>P{w+, bamP-GAL4-VP16} mnm$^{Z3-3298}$ /<br>P{v+, UAS-V20thr^{shmiR9} }attP2 mnm$^{Z3-5578}$
CID-EGFP His2Av-mRFP<br>Df/+ thr-RNAi mnm	w*/Y; {w+, gCid-EGFP-Cid}II.1 P{w+, His2Av-mRFP}II.2/<br>Df(2R)BSC338; P{w+, bamP-GAL4-VP16} mnm$^{Z3-3298}$ /<br>P{v+, UAS-V20thr^{shmiR9} }attP2 mnm$^{Z3-5578}$
CID-EGFP His2Av-mRFP<br>Df/+ thr-RNAi snm	w*/Y; {w+, gCid-EGFP-Cid}II.1 P{w+, His2Av-mRFP}II.2/<br>Df(2R)BSC338; P{w+, bamP-GAL4-VP16} snm$^{Z3-0317}$ /<br>snm$^{Z3-2138}$ P{v+, UAS-V20thr^{shmiR9} }attP2
EGFP-Tubulin His2Av-mRFP	w*/Y; P{w+, pUbi-EGFP-alphaTub84B}II P{w+, His2Av-mRFP}II.2/+;<br>P{w+, bamP-GAL4-VP16}/+
EGFP-Tubulin His2Av-mRFP<br>thr-RNAi	w*/Y; P{w+, pUbi-EGFP-alphaTub84B}II P{w+, His2Av-mRFP}II.2/+;<br>P{w+, bamP-GAL4-VP16}/P{v+, UAS-V20thr^{shmiR9} }attP2
