# Supplementary figures and images for: Separase Is Required for Homolog and Sister Disjunction during Drosophila melanogaster Male Meiosis, but Not for Biorientation of Sister Centromeres
Source: PLoS Genet. 2016 Apr 27;12(4):e1005996. doi: 10.1371/journal.pgen.1005996 (PMC4847790; doi:10.1371/journal.pgen.1005996)

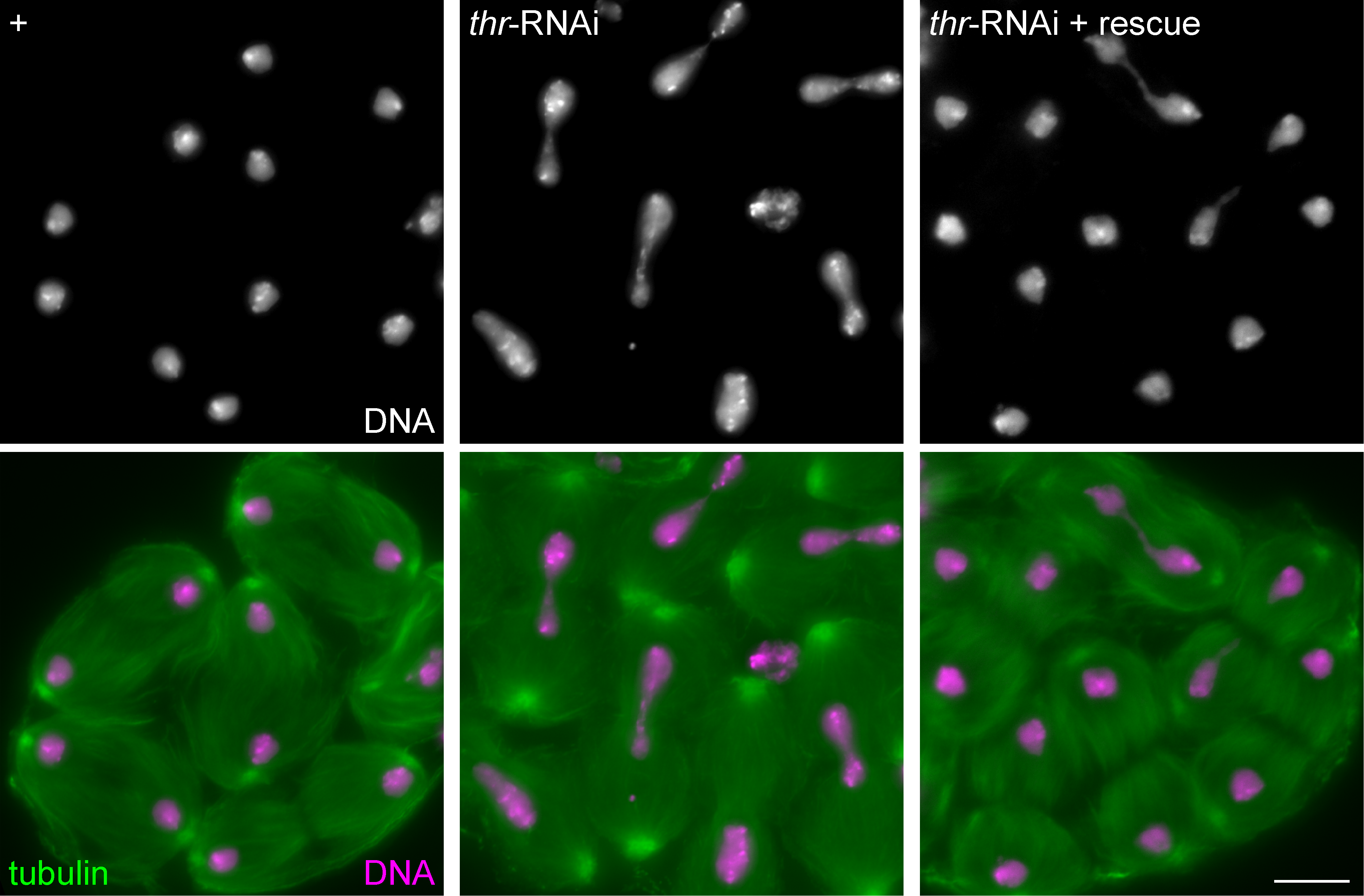

Supplement: S1 Fig — Squash preparations were labeled with anti-tubulin (tubulin) and a DNA stain (DNA). Testes were isolated from males without (+, left row) or with spermatocyte-specific THR depletion by transgenic RNAi (thr-RNAi, middle row), as well as from males in which spermatocyte-specific THR depletion was combined with expression of a thr transgene (UASt-thrRr) predicted to be RNAi-resistant as a result of silent mutations (thr-RNAi + rescue, right row). Precise genotype descriptions are given in S1 Table. The comparison of telophase I cysts reveals that thr-RNAi induces frequent and strong chromosome bridges which are strongly suppressed by the RNAi-resistant transgene. Scale bar = 10 μm. (TIF) [file pgen.1005996.s002.tif]

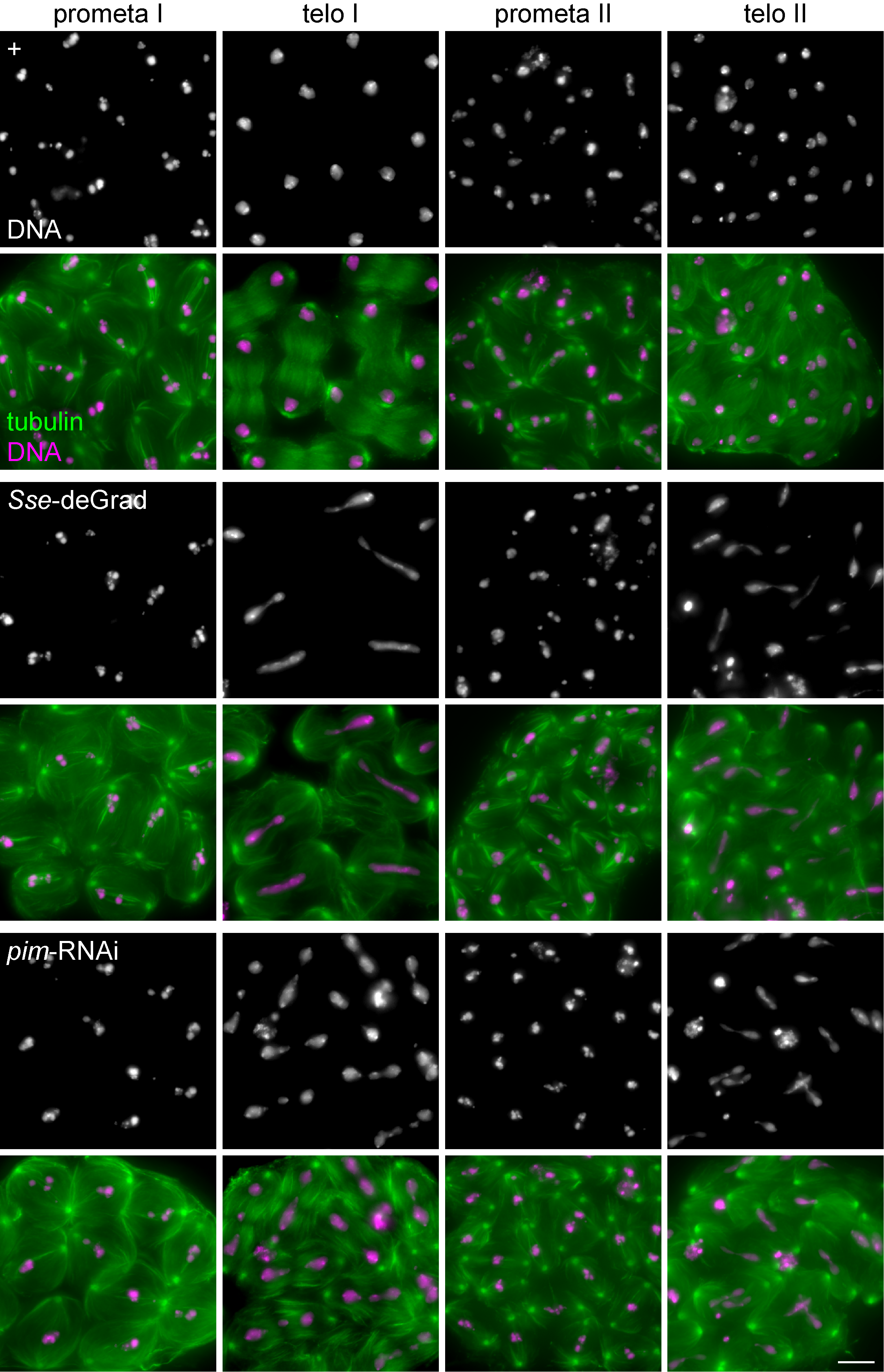

Supplement: S2 Fig — Squash preparations were labeled with anti-tubulin (tubulin) and a DNA stain (DNA). Testes were isolated from males without (+, upper panel) or with spermatocyte-specific SSE depletion by deGradFP (Sse-deGrad, middle panel), as well as from males with spermatocyte-specific PIM depletion by RNAi (pim-RNAi, lower panel). Precise genotype descriptions are given in S1 Table. The comparison of the cysts at the indicated meiotic stages reveals that SSE depletion induces chromosome bridges during meiosis I and II. Scale bar = 10 μm. (TIF) [file pgen.1005996.s003.tif]

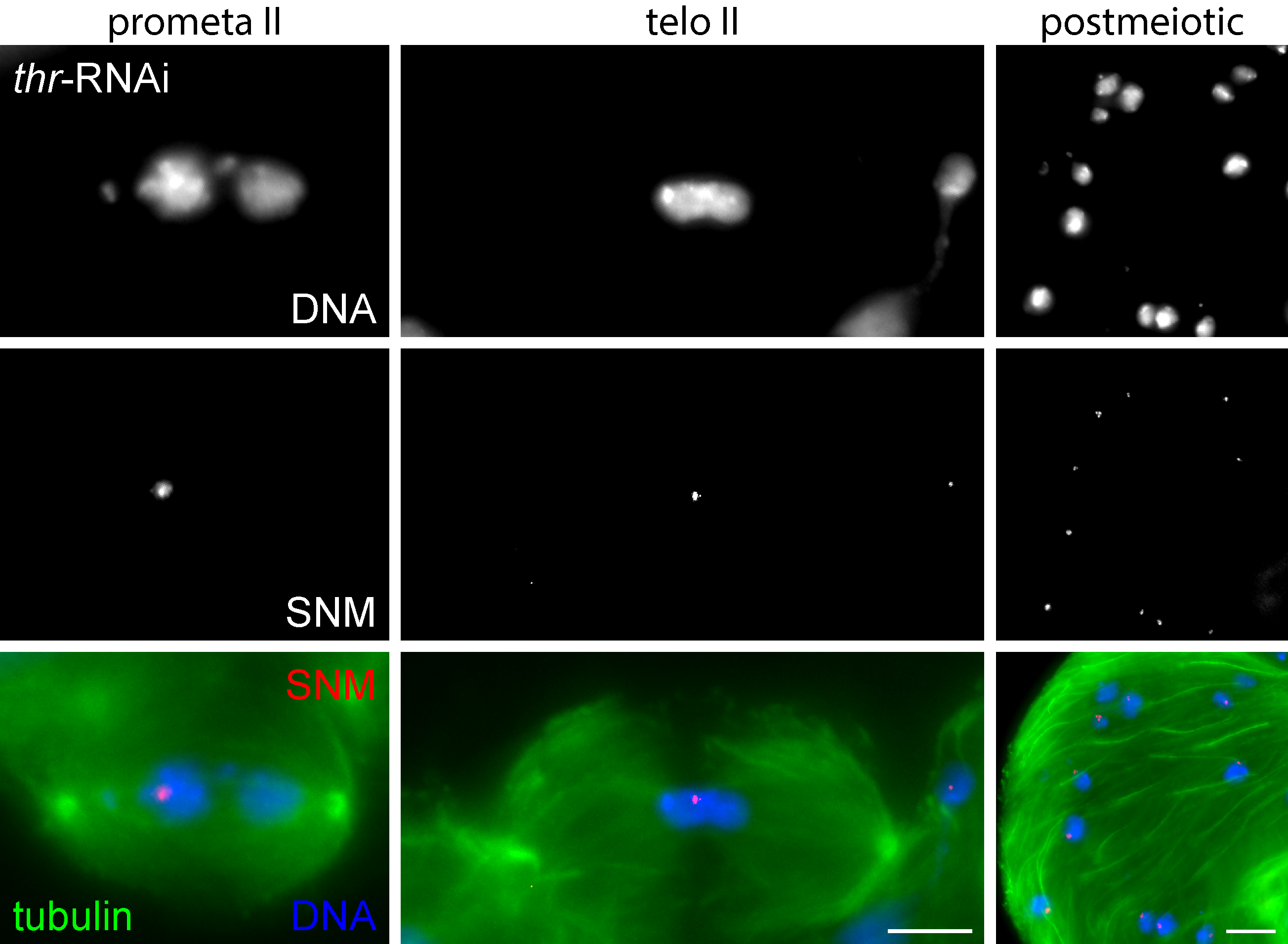

Supplement: S3 Fig — Squash preparations were labeled with anti-SNM (SNM), anti-tubulin (tubulin) and a DNA stain (DNA). Single spermatocytes at the indicated stages during meiosis II (scale bar = 5 μm) and part of a postmeiotic cyst (scale bar = 10 μm) are displayed. (TIF) [file pgen.1005996.s004.tif]

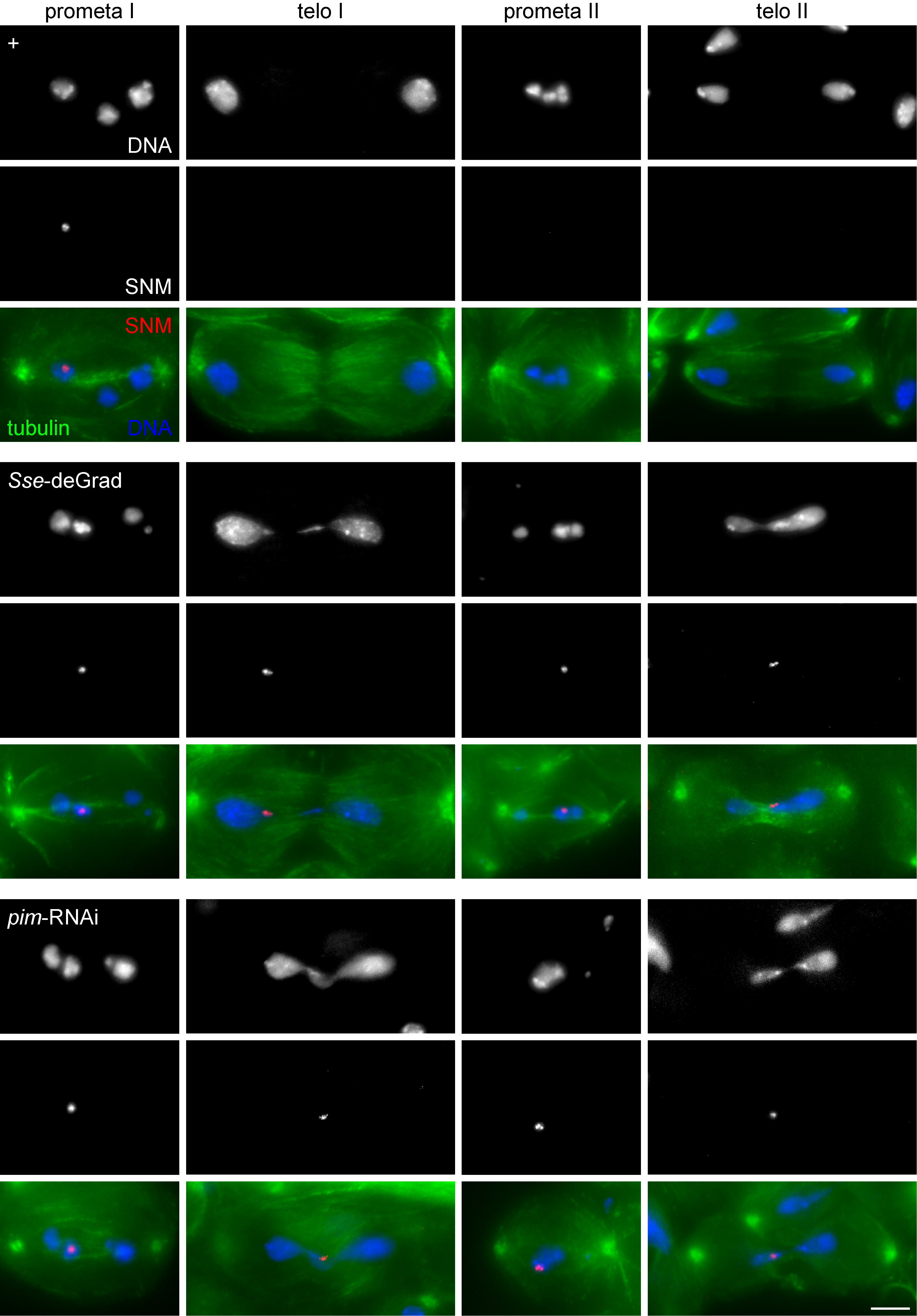

Supplement: S4 Fig — Testes were isolated from males without (+, upper panel) or with spermatocyte-specific SSE depletion by deGradFP (Sse-deGrad, middle panel), as well as from males with spermatocytes-specific PIM depletion by RNAi (pim-RNAi, lower panel). Squash preparations were labeled with anti-SNM (SNM), anti-tubulin (tubulin) and a DNA stain (DNA). Single spermatocytes at the indicated meiotic stages are displayed. Scale bar = 5 μm. (TIF) [file pgen.1005996.s005.tif]

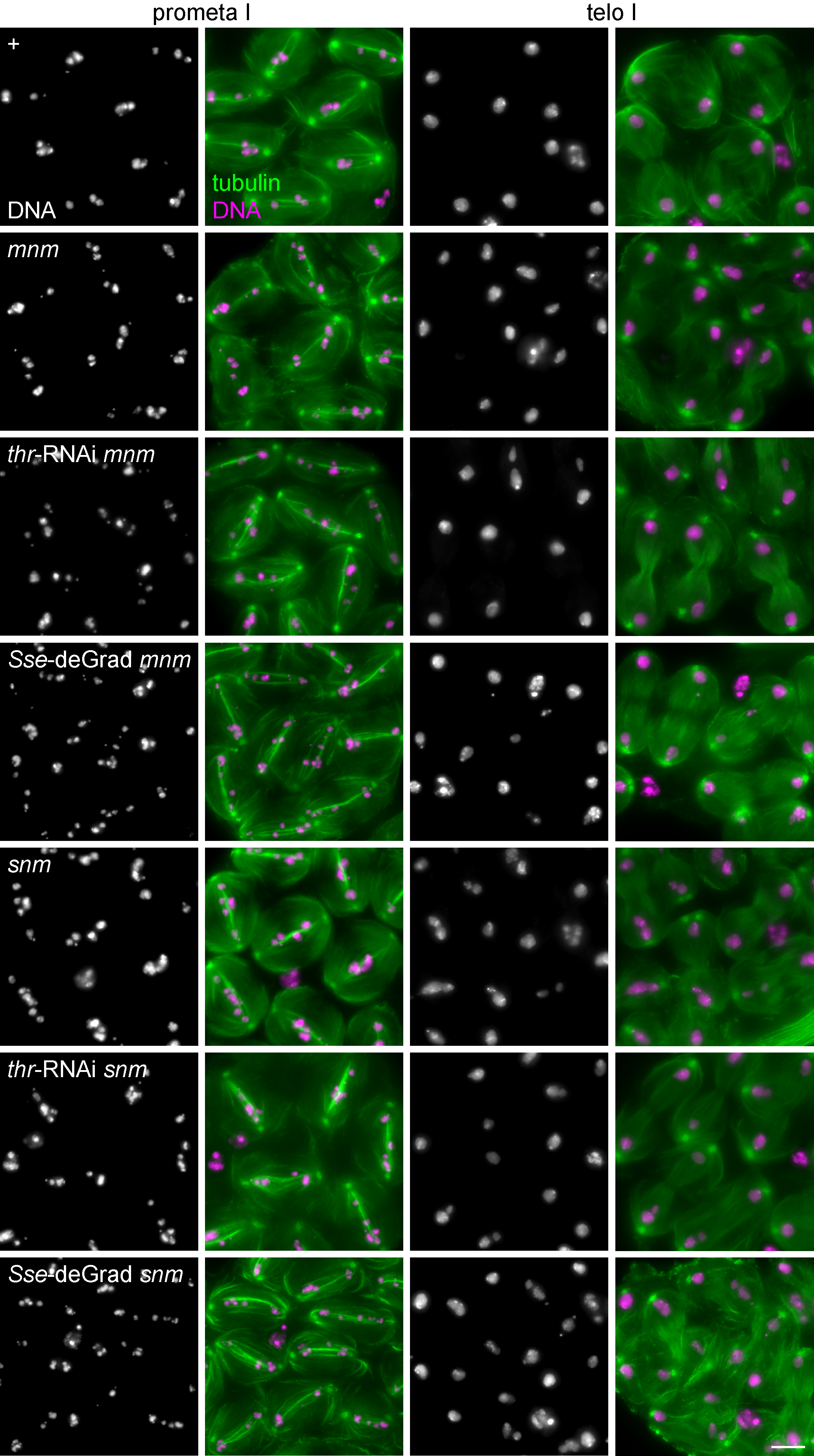

Supplement: S5 Fig — Squash preparations were labeled with anti-tubulin (tubulin) and a DNA stain (DNA). Testes were isolated from males with the indicated genotypes. + indicates absence of THR and SSE depletion as well as absence of mnm and snm mutations. mnm and snm indicate loss-of-function mutations. thr-RNAi and Sse-deGrad indicate spermatocyte-specific depletion with transgenic RNAi and deGradFP, respectively. Precise genotype descriptions are given in S1 Table. THR and SSE depletion do not induce bridges during meiosis I when the alternative homolog conjunction system does not function as a result of mutations in mnm or snm. Scale bar = 10 μm. (TIF) [file pgen.1005996.s006.tif]

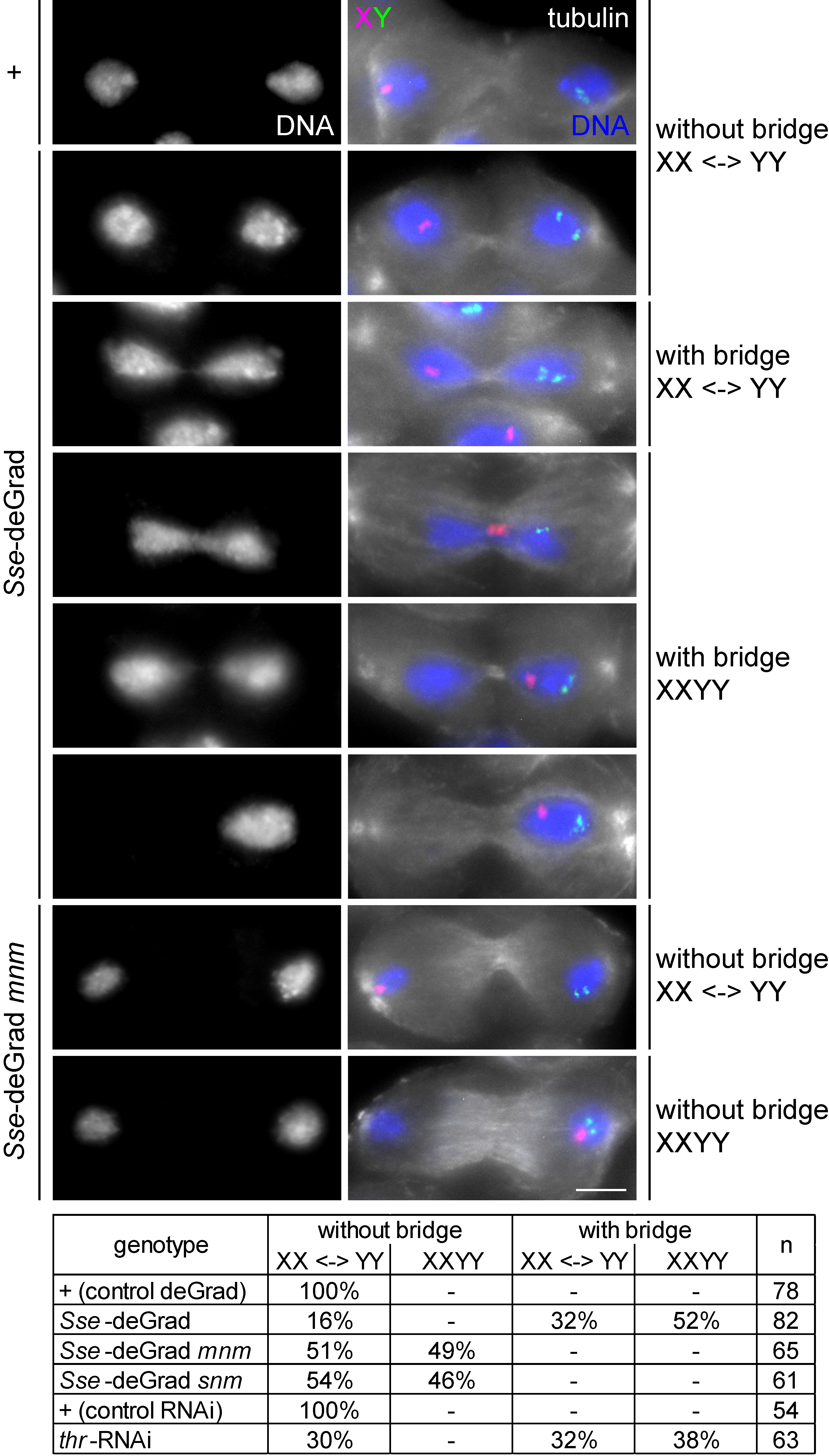

Supplement: S6 Fig — Squash preparations were labeled with anti-tubulin (tubulin) and a DNA stain (DNA). In addition, FISH with a red fluorescent X chromosome probe (X) and a green fluorescent Y chromosome probe (Y) was performed. As the X probe hybridizes close to the centromere (heterochromatic region h31), a single dot representing both sister chromatids is usually observed during meiosis I. In contrast, the Y probe which hybridizes to a region within the long arm (h6) usually generates two dots during meiosis I, one for each sister chromatid. The displayed single spermatocytes during telophase I were from males without (+) or with spermatocyte-specific SSE depletion by deGradFP (Sse-deGrad). In addition, mnm mutant males with identical spermatocyte-specific SSE depletion are displayed as well (Sse-deGrad mnm). In control (+), chromosome bridges were absent (without bridge) and X and Y were segregated into opposite daughter nuclei (XX<->YY). After Sse-deGrad, only very few telophase I figures were apparently normal, while the large majority had a chromosome bridge (with bridge) or complete separation failure which was also scored as bridged. In some cases, X and Y were on opposite sides of these bridges (XX<->YY). However, more frequently X and Y were not segregated apart (XXYY) and signals were observed at various positions along the bridge. Loss of mnm function was found to suppresses chromosome bridge induction by Sse-deGrad and was associated with random segregation of X and Y chromosomes into either the same daughter nucleus (XXYY) or into opposite daughter nuclei (XX<->YY). The frequency of these phenotypes observed in the different genotypes is given at the bottom in a table that includes data from THR depletion by transgenic RNAi. (TIF) [file pgen.1005996.s007.tif]

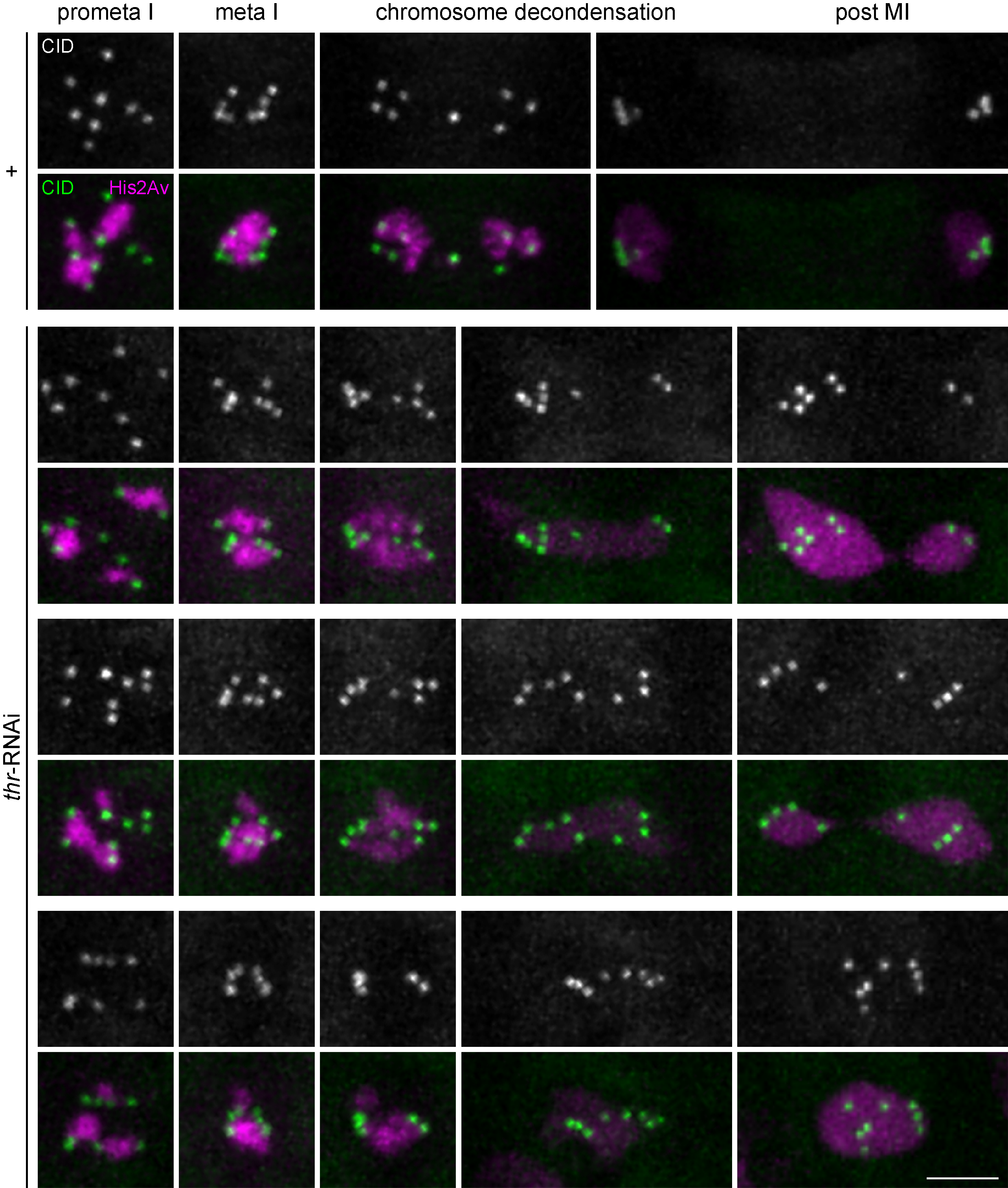

Supplement: S7 Fig — Live imaging performed with testes from cid-EGFP His2Av-mRFP males which have green fluorescent centromeres and red fluorescent chromatin. In addition, spermatocyte-specific THR depletion was either absent (+) or present (thr-RNAi). Still frames are presented from a representative control spermatocyte and three thr-RNAi spermatocytes during progression through meiosis I. The still frames were oriented so that the spindle axis is horizontal. Moreover, still frames where centromere number and positions were most clearly resolved during a particular meiotic stage were selected from a given movie. The second still frame always illustrates very late metaphase I. The first still frames illustrating prometaphase I were between 11 and 15 minutes before the late metaphase I frame. The third still frames (chromosome decondensation) were between five and six minutes after the late metaphase I frame. The fourth still frames (post MI) were between 14 and 20 minutes after the late metaphase I frame. In case of the three thr-RNAi spermatocytes, the fifth still frames were between 47 and 57 minutes after the late metaphase I frame, revealing bridging in two cases as well as complete separation failure in one case (bottom). While thr-RNAi prevents homolog separation, it interferes neither with the temporal dynamics of meiosis I progression nor with alignment of the bivalents into the metaphase I plate, indicating that spindles and kinetochores are functional. Scale bar = 5 μm. (TIF) [file pgen.1005996.s008.tif]

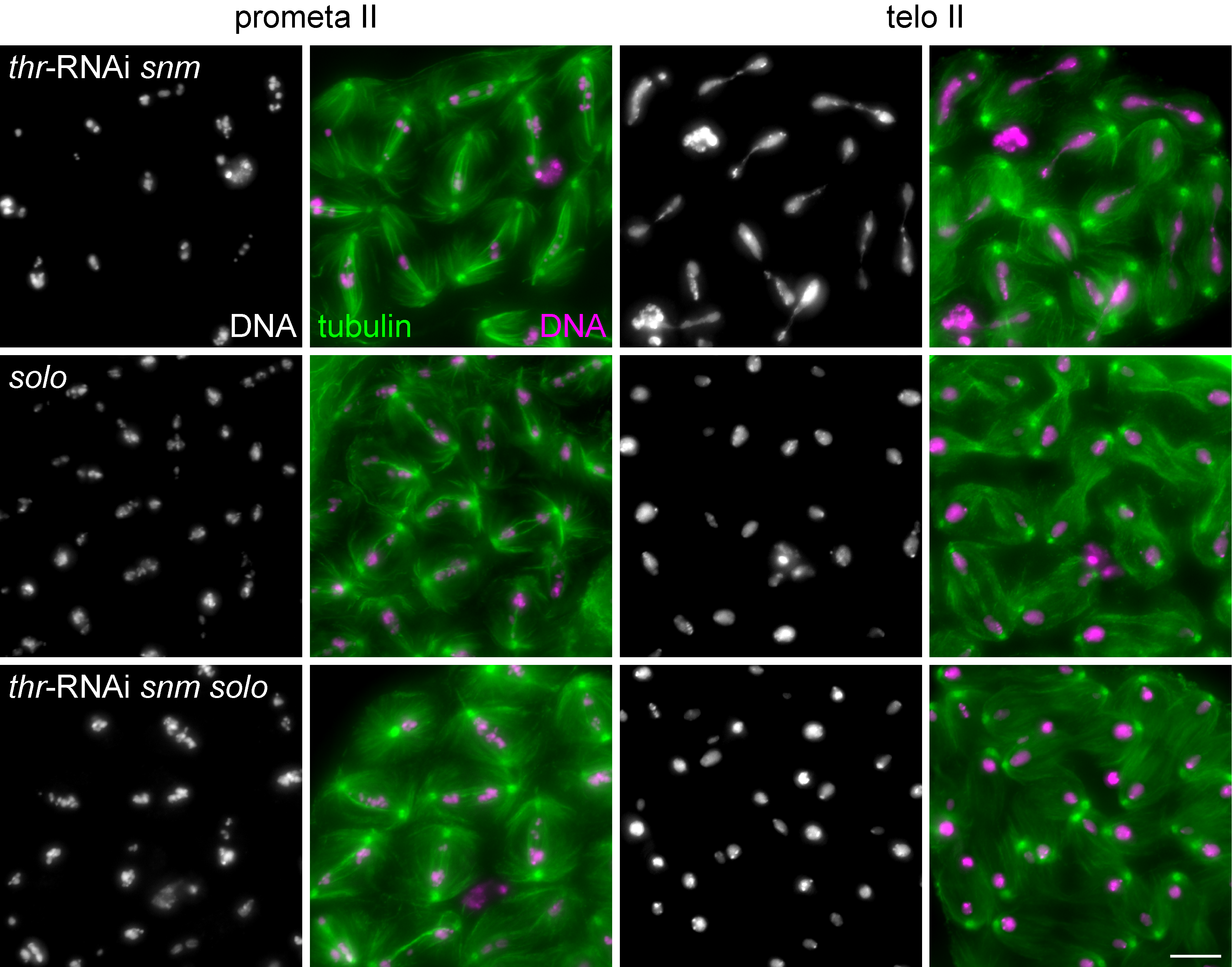

Supplement: S8 Fig — Squash preparations were labeled with anti-tubulin (tubulin) and a DNA stain (DNA). Testes were isolated from males with the indicated genotypes. thr-RNAi indicates spermatocyte-specific THR depletion by transgenic RNAi. solo and snm indicate loss-of-function mutations. Precise genotype descriptions are given in S1 Table. The comparison of the cysts at the indicated meiotic stages reveals that the chromosome bridges during telophase II which are observed after THR depletion in snm mutants, are no longer detectable when spermatocytes also lack solo function. Scale bar = 10 μm. (TIF) [file pgen.1005996.s009.tif]

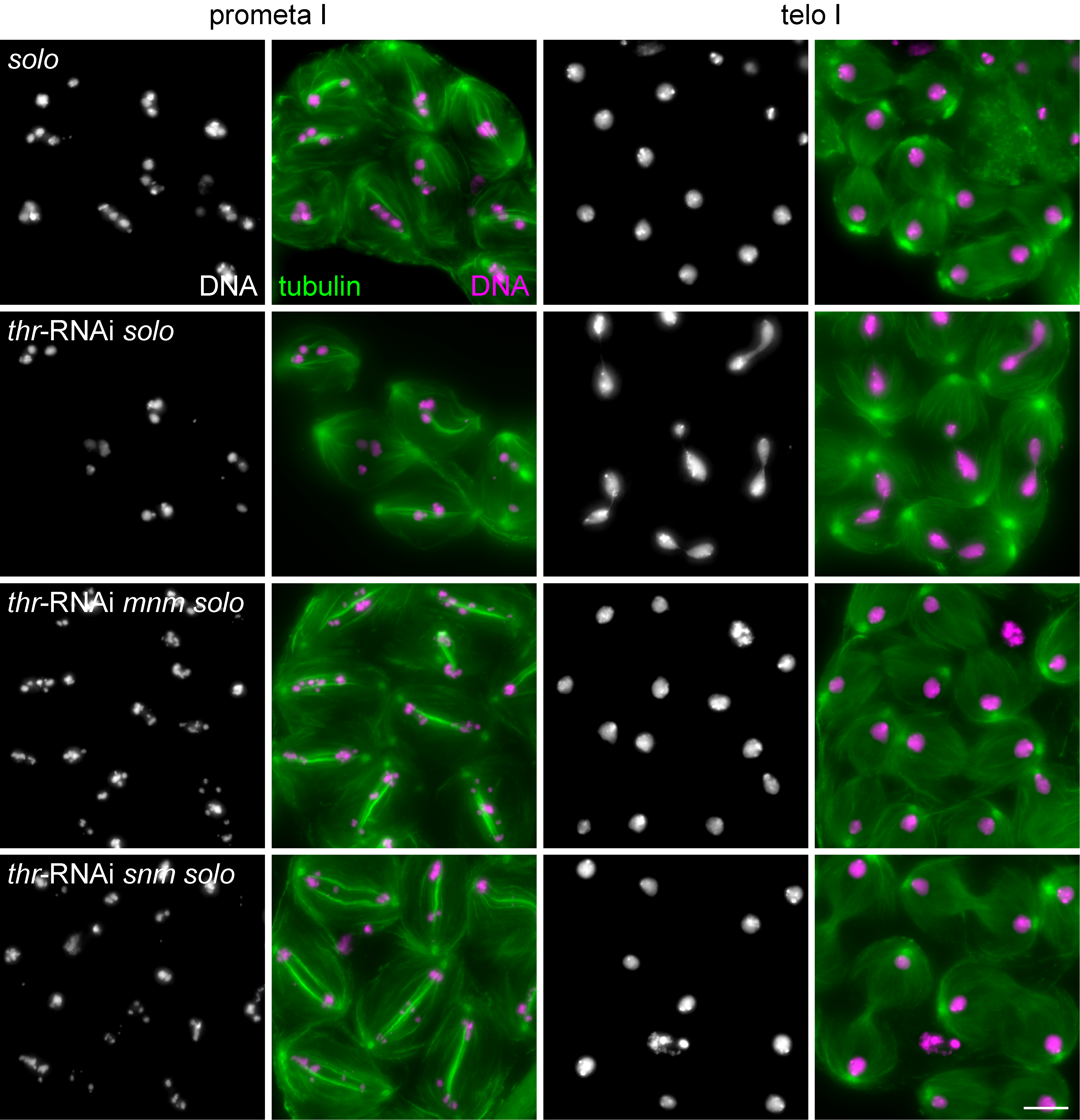

Supplement: S9 Fig — Squash preparations were labeled with anti-tubulin (tubulin) and a DNA stain (DNA). Testes were isolated from males with the indicated genotypes. thr-RNAi indicates spermatocyte-specific THR depletion by transgenic RNAi. solo, mnm and snm indicate loss-of-function mutations. Precise genotype descriptions are given in S1 Table. The comparison of the cysts at the indicated meiotic stages reveals that the chromosome bridges during telophase I, which are observed after THR depletion in solo mutants, are no longer detectable in spermatocytes which are unable to perform homolog conjunction due to lack of mnm or snm function. Scale bar = 10 μm. (TIF) [file pgen.1005996.s010.tif]

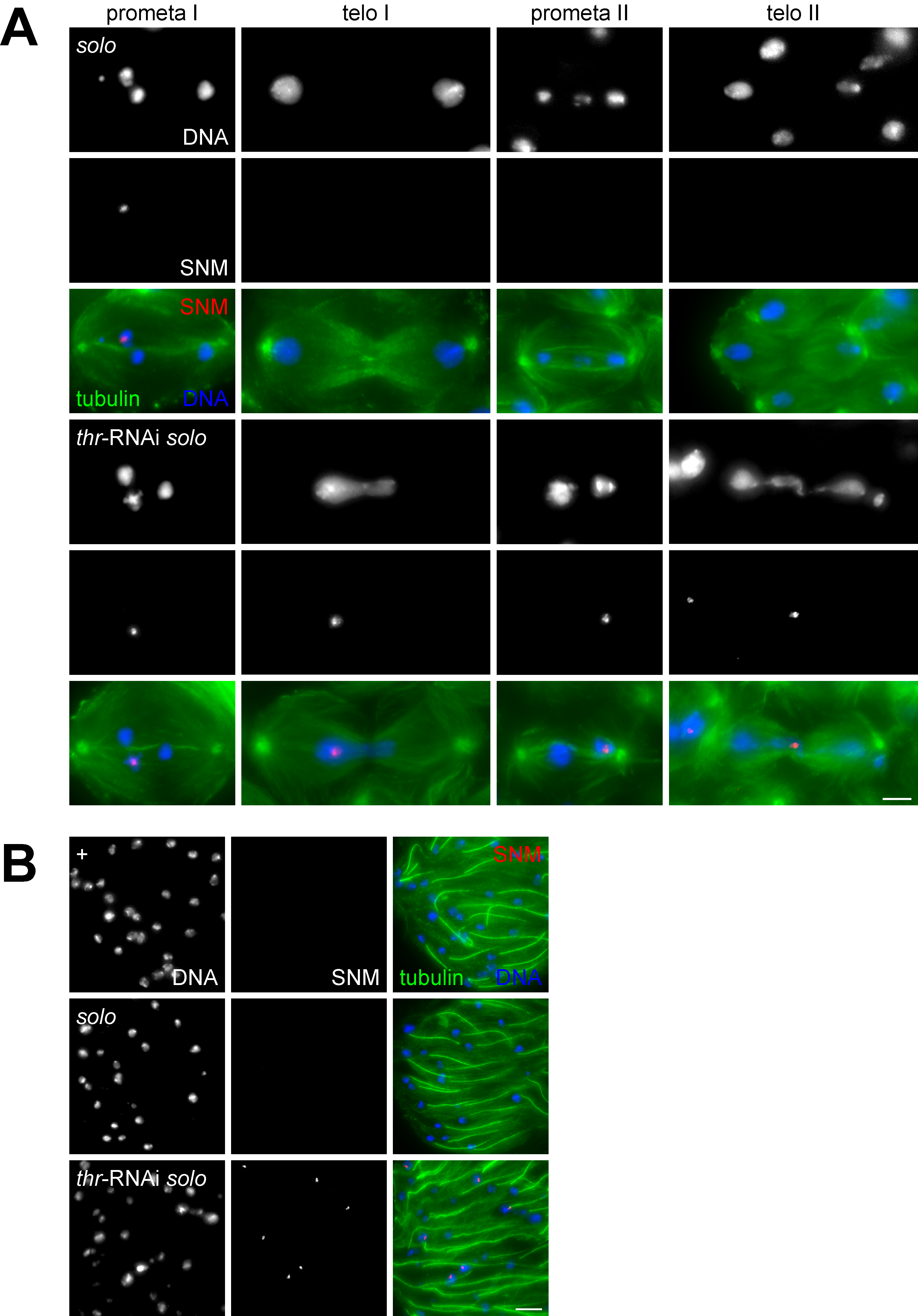

Supplement: S10 Fig — Testes were isolated from solo mutant males without (solo) or with spermatocyte-specific THR depletion (thr-RNAi solo), as well as from males without spermatocyte-specific THR depletion (+). Squash preparations were labeled with anti-SNM (SNM), anti-tubulin (tubulin) and a DNA stain (DNA). Single spermatocytes at the indicated stages (A, scale bar = 5 μm) and part of a postmeiotic cyst (B, scale bar = 10 μm) are displayed. (TIF) [file pgen.1005996.s011.tif]
